# Evaluating deep learning time series models for PM_2.5_ forecasting across diverse horizons

**DOI:** 10.1016/j.isci.2026.114770

**Published:** 2026-01-21

**Authors:** Ling Zeng, Runan Dong, Meng Yuan, Linhai Jing, Shoutao Jiao

**Affiliations:** 1 Geomathematics Key Laboratory of Sichuan Province, Chengdu Technological University, Chengdu 610059, China; 2 College of Mathematical Science, Chengdu Technological University, Chengdu 610059, China; 3 School of Artificial Intelligence, China University of Geosciences, Beijing 100083, China; 4 China Geological Survey Natural Resources Comprehensive Survey Command Center, Beijing 100055, China

**Keywords:** Atmospheric science, Atmospheric chemistry, Atmosphere modelling, Environmental science, Environmental health, Pollution, Machine learning

## Abstract

Air pollution, particularly PM2.5, poses a major health challenge in urban areas such as Chengdu, China, where basin topography and intense emission sources exacerbate pollutant concentrations. This study evaluates four deep-learning time series algorithms—LSTM, CNN-LSTM, Transformer, and Transformer-LSTM—for PM2.5 forecasting, comparing univariate and four multivariate configurations incorporating auxiliary pollutants (CO, NO_2_, O_3_, SO_2_) and meteorological factors (temperature, pressure, precipitation, wind speed). Using two years of daily data (November 2022–October 2024), models are assessed across monthly, seasonal, half-year, and annual horizons with complete and incomplete seasonal datasets. Results demonstrate: Transformer-LSTM yields superior performance with higher R^2^ and lower MAE% and RMSE%, especially when augmented by meteorological factors over pollutants; complete seasonal training improves performance, while gaps exceeding three months between training and prediction reduce reliability due to evolving PM2.5 dynamics. These findings underscore meteorological integration, data-driven modeling, seasonal completeness, and timely prediction for pollution control for policymakers in Chengdu.

## Highlights

• Transformer-LSTM delivers the best PM2.5 prediction across horizons in Chengdu
• Meteorology, especially temperature, outweighs auxiliary pollutants
• Complete seasonal data enhances prediction performance
• Three-month gaps between training and prediction reduce reliability

## Graphical abstract

**Figure:**
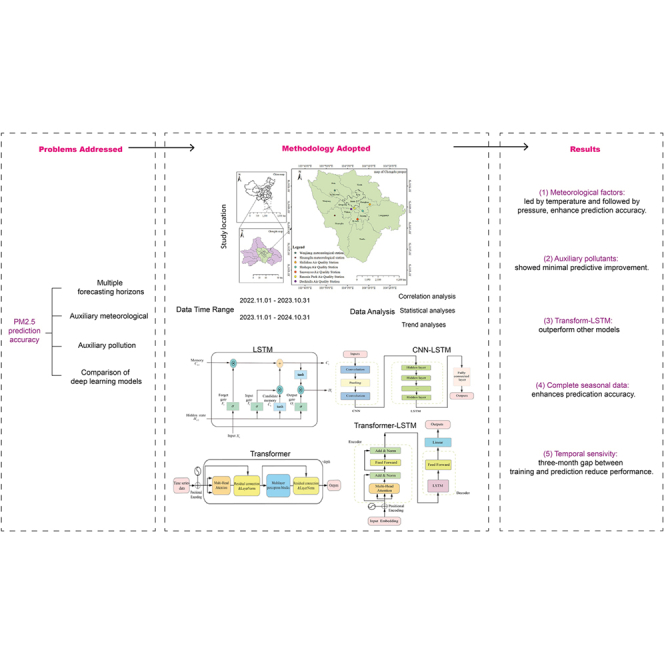

## Introduction

Air pollution, particularly PM_2.5_ (particles ≤2.5 μm), poses a pressing global urban challenge due to its deep lung penetration and links to severe respiratory and cardiovascular diseases.^1^^,^^2^ The World Health Organization deems concentrations above 35 μg/m^3^ hazardous, raising growing concerns about public health. Chengdu, a major city in southwestern China’s Sichuan Basin, faces severe PM_2.5_ pollution. The basin’s topography—low wind speeds, temperature inversions, and high humidity—traps pollutants, worsening air quality.^3^^,^^4^ Dense population, industrial emissions, shallow mixing layers, and winter thermal inversions drive frequent pollution episodes.^5^^,^^6^^,^^7^ Nitrates, a key secondary aerosol, account for nearly half of Chengdu’s PM_2.5_.^8^^,^^9^

Recent predictive PM_2.5_ models evolved from traditional statistical methods and machine learning to deep learning architecture.^10^^,^^11^^,^^12^^,^^13^^,^^14^^,^^15^^,^^16^^,^^17^ **Traditional statistical models,** such as regression models and autoregressive integrated moving average (ARIMA), provide interpretable results but fail to capture the complex temporal dependencies inherent in PM_2.5_ time-series data.^18^^,^^19^ **Machine learning methods,** such as random forests and support vector machines, boost accuracy but lack scalability and generalization across diverse datasets.^20^^,^^21^^,^^22^ Outperforming these, **deep learning-based methods,** such as convolutional neural networks (CNNs), long short-term memory (LSTM) networks, and Transformers, excel in nonlinear modeling and large datasets.^23^ CNNs excel at capturing PM_2.5_ spatial features but struggle with temporal dependencies and high computation.^24^ LSTMs suit sequential data and time-series forecasting, yet struggle with vanishing gradients over long sequences.^25^ Transformers utilize attention mechanisms to effectively handle long-range dependencies, but are computationally demanding.^26^ Hybrid models such as CNN-LSTM and Transformer-LSTM, combine these strengths to enhance spatial-temporal modeling.^27^^,^^28^^,^^29^ Transformer-LSTM, though underexplored for PM_2_._5_ prediction, integrates Transformer’s self-attention with LSTM’s sequential capabilities, improving short- and long-term trend capture.^26^^,^^30^ Its practical applications are limited, needing more research.

In addition to predictive modeling techniques, researchers have also explored different input frameworks, including univariate (PM_2.5_-only) and multivariate (incorporating auxiliary meteorological factors) approaches. Numerous studies have shown that incorporating auxiliary meteorological variables—such as temperature, humidity, and wind speed—can improve prediction accuracy for PM_2.5_.^31^^,^^32^ However, the incorporation of co-pollutants such as CO, NO_2_, SO_2_, and O_3_ as auxiliary variables remains underexplored, despite their demonstrated correlations with PM_2.5_. Furthermore, few studies have systematically compared the performance of univariate (PM_2.5_-only) and multivariate models within the same deep learning architecture. This gap limits a comprehensive understanding of how auxiliary co-pollutants contribute to predictive accuracy and under what conditions they offer the greatest benefit.^33^

An underexplored aspect in PM_2.5_ prediction is how model performance varies across different forecasting horizons when using training datasets of varying completeness and duration. Many existing studies focus on predicting PM_2.5_ over specific time spans without systematically examining how the length and composition of training data affect predictions for diverse forecasting periods, such as one month, one season, half a year, or a full year. Short-term forecasts (e.g., monthly or seasonal predictions) are vital for real-time air quality monitoring and emergency response, while long-term forecasts (e.g., half-yearly or annual predictions) are critical for environmental policy planning and assessment.^34^ Assessing the sensitivity of predictive models to different forecasting horizons, training data completeness, and the temporal gap between training and prediction periods is essential to support applications ranging from immediate air quality management to long-term environmental strategies.

This study aims to address these gaps by: (1) evaluating the performance of Transformer-LSTM against other deep learning models (LSTM, CNN-LSTM, and Transformer), (2) systematically investigating the sensitivity of these models to varying forecasting horizons using training datasets of different completeness and duration, and (3) comparing univariate (PM_2.5_-only) with multivariate models within the same deep learning architectures, by incorporating auxiliary pollutants (O_3_, NO_2_ SO_2_, and CO) to investigate their predictive effect on PM_2.5_, while also evaluating the predictive contributions of meteorological factors (temperature, pressure, precipitation, wind speed). Using real-world data from Chengdu, this study reveals the predictive role of auxiliary pollutants and meteorological variables, the efficacy of hybrid architectures, and the impact of forecasting horizon, training data completeness, and duration on PM_2.5_ prediction accuracy, aiding air quality prediction and policy.

## Results

This study evaluates four deep-learning methods—LSTM, CNN-LSTM, Transformer, and Transformer-LSTM—for predicting PM_2_._5_ trends in Chengdu. Each method is tested in univariate (PM_2_._5_ only) and multivariate configurations, incorporating auxiliary variables in four multivariate configurations: (1) CO and NO_2_, (2) CO only, (3) O_3_ and SO_2_, and (4) meteorological factors (temperature, pressure, precipitation, wind speed), as detailed in Section “configurations of auxiliary variables.” This yields 20 models (four methods × five configurations) assessed across multiple forecasting horizons. Time-sensitivity analyses are divided into two categories: Category 1 uses complete yearly four-season data to predict the next year’s long-term (full-year), mid-term (half-year), seasonal, and monthly trends; Category 2 uses incomplete seasonal data to predict remaining months within the same year (see Section “setups of time-sensitivity analyses”).

Model performance is evaluated using MAE%, RMSE%, and *R*^2^ (see Section “model evaluation metrics”), which normalize errors relative to average PM_2_._5_ values for consistent comparisons across models and scenarios.

### Configurations of auxiliary variables

To evaluate the contribution of auxiliary variables to PM_2_._5_ forecasting, pollutants (CO, NO_2_, O_3_, and SO_2_) and meteorological factors (temperature, pressure, precipitation, and wind speed) were organized into four configurations. These configurations assess the predictive impact of each group and compare their performance against univariate (PM_2_._5_-only) models, as detailed later in discussion.

#### Pollutant configuration 1: and NO_2_

This configuration includes CO and NO_2_, selected for their high correlations with PM_2_._5_ (0.788 and 0.727, respectively). The objective is to determine whether these strongly correlated pollutants enhance PM_2_._5_ prediction accuracy compared to univariate models and to evaluate their contribution to forecasting.

#### Pollutant configuration 2: CO only

CO and NO_2_ in pollutant configuration 1 exhibit strong intercorrelation (0.747), which may introduce covariance and reduce model stability. This configuration uses only CO, which has the highest correlation with PM_2_._5_ (0.788), to isolate its predictive impact. The goal is to compare its performance against Configuration 1 to assess whether covariance between CO and NO_2_ affects prediction accuracy.

#### Pollutant configuration 3: O_3_ and SO_2_

This configuration includes O_3_ and SO_2_, with moderate correlations to PM_2_._5_ (0.294 and 0.205, respectively). The aim is to evaluate whether these less correlated pollutants improve PM_2_._5_ prediction over univariate models and to assess their contribution to forecasting accuracy.

#### Meteorological configuration: temperature, pressure, precipitation, and wind speed

This configuration combines four meteorological factors—temperature, pressure, precipitation, and wind speed—with correlations to PM_2_._5_ ranging from 0.257 to 0.491. The objective is to evaluate their collective predictive impact on PM_2_._5_ forecasting and compare their performance against pollutant configurations and univariate models.

### Setups of time-sensitivity analyses

Time-sensitivity analyses were structured into two main categories: **predictions using complete yearly four-season data** and **predictions using incomplete seasonal data**. Each category was further divided based on forecasting horizons, as outlined later in discussion.

#### Category 1: Predictions using complete yearly four-season data

This category evaluates model performance using a full year of four-season data (November 2022 to October 2023) for training, with predictions tested on the following year (November 2023 to October 2024) across various horizons. This setup captures complete seasonal patterns, enabling robust accuracy analysis over diverse time frames (Table 1).

**Table 1:** Subcategories and cases for Category 1

Category 1′	Case	Description	Time Period
**Category 1-1:** **Long-term predictions**	Full year forecast	Forecast for an entire year	November 2023 – October 2024
**Category 1–2:** **Mid-term half-year predictions**	Case 1	Predict winter and spring	November 2023 – April 2024
	Case 2	Predict summer and autumn	May 2024 – October 2024
**Category 1–3:** **Short-to-medium-term seasonal predictions**	Case 1	Predict winter	November 2023 – January 2024
	Case 2	Predict spring	February 2024 – April 2024
	Case 3	Predict summer	May 2024 – July 2024
	Case 4	Predict autumn	August 2024 – October 2024
**Category 1–4:** **Short-term monthly predictions**	Case 1	Predict November 2023	November 2023
	Case 2	Predict December 2023	December 2023
	Case 3	Predict January 2024	January 2024
	Case 4	Predict February 2024	February 2024
	Case 5	Predict March 2024	March 2024
	Case 6	Predict April 2024	April 2024
	Case 7	Predict May 2024	May 2024
	Case 8	Predict June 2024	June 2024
	Case 9	Predict July 2024	July 2024
	Case 10	Predict August 2024	August 2024
	Case 11	Predict September 2024	September 2024
	Case 12	Predict October 2024	October 2024

#### Category 2: Predictions using incomplete yearly season data

This category tests models trained on partial yearly data (November 2023 to October 2024) to predict remaining months within the same cycle, maintaining a full-year span. It assesses forecasting efficacy with limited seasonal input, focusing on later-year trends (Table 2).

**Table 2:** Subcategories and cases for Category 2 PM_2.5_ forecasting

Category 1	Case	Description	Training Period	Prediction Period
**Category 2-1:** **Seasonal gap predictions**	Case 1	Train in winter and spring to predict summer and autumn.	November 2023 to April 2024	May 2024 to October 2024
	Case 2	Train on winter, spring, and summer to predict autumn.	November 2023 to July 2024	August 2024 to October 2024
**Category 2-2:** **Short-term predictions with missing months**	Case 1	Train on the first 10 months to predict the next 2 months.	November 2023 to August 2024	September 2024 to October 2024
	Case 2	Train on the first 11 months to predict the final month.	November 2023 to September 2024	October 2024

### Results of models

This study evaluates 460 models derived from four deep-learning methods (LSTM, Transformer, CNN-LSTM, Transformer-LSTM), five prediction configurations (one univariate and four multivariate configurations), and 23 forecasting horizons from time-sensitivity analyses. Predictive performance is summarized through trends (Figures S1–S23) and metrics (Tables S1–S6) in the supplemental information, using MAE%, RMSE%, and *R*^2^.

#### Prediction trends

Figures S1–S23 align with the forecasting horizons outlined in Section “setups of time-sensitivity analyses,” each with four subplots: (a) LSTM, (b) Transformer, (c) CNN-LSTM, and (d) Transformer-LSTM. Subplots display observed PM_2_._5_ (black solid line), alongside predictions, distinguished by line styles and colors: orange solid for univariate, blue dashed for the CO + NO_2_ multivariate configuration, purple dashed for the CO-only configuration, green dashed for the SO_2_+O_3_ configuration, and red dashed for the four meteorological factor configuration (temperature, pressure, precipitation, wind speed). Figures S1–S19 (Category 1) are based on complete yearly four-season data, while Figures S20–S23 (Category 2) utilize incomplete seasonal data.

① Figure S1: Long-term forecast (next year).
② Figures S2 and S3: Mid-term forecasts (first and second half-years).
③ Figures S4–S7: Short-to-medium-term seasonal forecasts (Winter, Spring, Summer, Autumn).
④ Figures S8–S19: Short-term monthly forecasts (Nov 2023–Oct 2024).
⑤ Figures S20 and S21: Seasonal gap predictions (initial seasons to rest).
⑥ Figures S22 and S23: Short-term forecasts with missing months (final 1–2 months).

For Category 1 (S1 to S19), Figures S1, S2, and S4 show the best fit to observed PM_2_._5_ trends, followed by S8 to S10 with strong alignment, particularly when meteorological factors are included. Other figures struggle with significant fluctuations. In Category 2 (S20 to S23), accuracy declines overall, but Figures S21 and S22, particularly with the Transformer-LSTM model incorporating meteorological data, better capture trends. Models trained on complete yearly data, enhanced by meteorological insights, consistently surpass those relying on incomplete seasonal data in predictive accuracy.

#### Performance metrics

The full performance metrics across all cases are listed in Tables S1–S6. Tables S1–S4 cover Category 1 (complete yearly four-season data), while Tables S5 to S6 address Category 2 (incomplete yearly data).

**In Category 1:** Table S1 (long-term prediction) exhibits the highest overall performance. The multivariate Transformer-LSTM model, incorporating four meteorological factors, achieves the greatest accuracy, as evidenced by the highest *R*^2^ values and the lowest error metrics. Closely following, Case 1 in Table S2 (mid-term prediction for the first half-year) demonstrates robust performance, with the multivariate Transformer-LSTM model leveraging meteorological factors performing particularly well. For seasonal predictions in Table S3, Case 1 (winter) yields the best results, with the multivariate Transformer-LSTM model utilizing meteorological factors achieving superior performance, likely attributable to stronger temporal correlations with prior data. In Table S4 (monthly predictions), performance declines across Cases 3 (January), 2 (December), and 1 (November). The multivariate Transformer-LSTM model with meteorological factors leads in Cases 3 and 2, but is less effective compared to the winter season results in Table S3.

**In Category 2:** Tables S5 and S6 exhibit diminished performance relative to Category 1, marked by lower *R*^2^values and higher RMSE and MAE metrics. In Table S5, Case 2 demonstrates relatively stable performance compared to the other cases within the table, while in Table S6, Case 1 shows similar relative stability. Moreover, the multivariate Transformer-LSTM incorporating meteorological factors outperforms other models in both tables, yielding slightly higher *R*^2^ and reduced error metrics compared to its univariate counterpart or models with alternative auxiliary variables. Although incomplete data constrain overall accuracy, the inclusion of meteorological factors enhances model resilience.

## Discussion

### Sensitivity for the time gap between training and the prediction period

The analysis of sensitivity to the time gap between training and prediction periods is relevant only when using complete training data (Category 1, Tables S1–S4), as incomplete data predictions (Category 2, Tables S5 and S6) do not consider this gap. Analysis of Tables S1–S4 reveals the following.

① Predictions immediately following the training period exhibited relatively high and stable *R*^2^ values. This includes long-term predictions (Table S1 Case 1, immediately adjacent to the training period), mid-term predictions for the first half-year (Table S2, Case 1, immediately adjacent to the training period), and short-to-medium-term predictions for the first quarter (Table S3, Case 1, immediately adjacent to the training period), and short-term monthly predictions for the first three months (Table S4, Cases 1–3, immediately adjacent to the training period).
② As the time gap between the training and prediction periods increased, model fit metrics, such as *R*^2^, declined significantly. For example, mid-term predictions for the second half-year (Table S2, Case 2) showed a marked decrease in *R*^2^. In some cases, *R*^2^ values even turned negative, as observed in short-to-medium-term predictions for the second, third, and fourth quarters (Table S3 Cases 2–4) and in short-term monthly predictions for months beyond the first three (Table S4 Cases 4–12). Notably, this decline becomes particularly pronounced when the temporal gap exceeds three months (one-quarter), indicating a critical threshold for maintaining predictive accuracy.

The decline in performance indicated above, associated with increasing time gaps between training and prediction periods, may be attributed to the difficulty in capturing complete temporal patterns, as training data becomes less representative of evolving PM_2_._5_ dynamics, such as seasonal shifts or new pollution sources, which are inadequately reflected beyond a three-month interval.

### Impact of completeness of training data

Category 1 (complete four-season training data) outperforms Category 2 (incomplete yearly data), as evidenced by Tables S1–S4 vs. Tables S5 and S6 and Figures S1–S19 vs. Figures S20–S23. Category 1 excels, especially for predictions immediately following training. This indicates that complete seasonal data enhances forecast accuracy, while incomplete data in Category 2 fails to capture seasonal patterns, lowering performance.

### Impact of training proportion in incomplete seasonal data

Tables S5 and S6, based on incomplete seasonal data from November 2023 to October 2024, reveal that a higher training data proportion generally boosts performance. Table S6, Case 1 (10 months training, the next 2 months prediction) outperforms Table S5, Case 2 (9 months training, 3 months prediction), followed by Table S5, Case 1 (6 months training, 6 months prediction). However, this trend breaks when predicting just one month: Table S6, Case 2 (11 months training, the next one-month prediction) shows reduced *R*^2^, suggesting excessive training data relative to the short one-month prediction horizon can lead to overfitting and yield diminishing returns.

### Challenges in single-month predictions immediately after the training period

Predicting a single month right after the training period proves challenging, as seen in Table S6, Case 2 (11 months of incomplete seasonal data) and Table S4, Case 1 (complete yearly seasonal data). Section “impact of training proportion in incomplete seasonal data” notes that increasing training data proportion typically enhances prediction accuracy. However, this trend reverses in Table S6, Case 2, where 11 months of training data for predicting the final month (Oct 2024) yields poorer performance. Similarly, Table S4, Case 1 (predicting Nov 2023) underperforms compared to Cases 2 and 3 (predicting Dec 2023 and Jan 2024, with a one-month gap).

This reduced accuracy may stem from.

① Limited time window: Predicting immediately after training leaves the model with a narrow time frame, hindering its ability to capture subtle PM_2_._5_ fluctuations just beyond the data.
② Insufficient time to understand trends: A small gap (e.g., one to two months, as in Table S4, Cases 2–3) allows the model to better identify longer-term trends, enhancing accuracy.

### Comparative performance of univariate, pollutant-based, and meteorology-based prediction

We evaluated 92 groups of univariate, pollutant-based, and meteorology-based models across 23 forecasting horizons using four deep-learning algorithms. Results indicate that only the meteorological factors configuration (temperature, pressure, precipitation, wind speed) consistently enhanced prediction accuracy over univariate models, yielding higher *R*^2^ and lower MAE% and RMSE%. In contrast, the three pollutant-based configurations (CO + NO_2_, CO-only, O_3_+SO_2_) showed no significant accuracy improvements over univariate models, though rare exceptions occurred with specific algorithms.

Specifically, in the first four subsections of the “discussion,” we evaluated stable cases across two categories: Category 1 includes long-term one-year predictions (Table S1, Case 1), mid-term first half-year predictions (Table S2, Case 1), short-to-medium-term first-quarter predictions (Table S3, Case 1), and short-term monthly predictions for the first three months (Table S4, Cases 1–3); Category 2 covers predictions using 9 months of training for 3 months (Table S5, Case 2) and 10 months for 2 months (Table S6, Case 1), which demonstrated relative stability but lower accuracy compared to Category 1.

Analysis of these stable cases indicates that CO-only predictions consistently showed slightly lower accuracy than CO + NO_2_, except in short-term monthly predictions (Table S4, Cases 1–3), where their performance was comparable with no consistent advantage for either. The O_3_+SO_2_ configuration yielded inconsistent results across all cases, showing no reliable pattern of improvement.

Additionally, across these stable cases, the four deep-learning architectures demonstrated consistent and significant improvements when incorporating meteorological features relative to their univariate counterparts.

Specifically, For LSTM, meteorological factors yielded modest but consistent gains, with Δ*R*^2^ ranging from 0.08 to 0.12 in Category 1 long-term (Table S1, Case 1) and mid-term first half-year (Table S2, Case 1) forecasts (e.g., RMSE% reduction of 12–18%), reflecting its foundational sequential modeling but limited capacity to leverage causal dispersion effects such as wind speed and precipitation without advanced feature extraction.

CNN-LSTM demonstrated the most pronounced relative improvements among the non-hybrid Transformer models, particularly in Category 2 incomplete data scenarios (e.g., Δ*R*^2^ up to 0.15 and 20–25% RMSE% reductions in 9-month training cases). This suggests that the convolutional layers effectively capture local meteorological patterns (e.g., temperature inversions), amplifying the hybrid’s sensitivity to external features and occasionally surpassing Transformer-LSTM’s absolute gains in short-term predictions.

The Transformer architecture benefited moderately from meteorological inputs (Δ*R*^2^ of 0.10–0.14; MAE% reductions of 15–22%), with stronger performance in Category 1 seasonal forecasts due to its attention mechanism prioritizing long-range dependencies influenced by pressure and temperature. However, these gains were less resilient in Category 2 compared to hybrids, highlighting potential attention dilution with incomplete seasonal data.

In contrast to these architectures, the Transformer-LSTM hybrid maintained the highest absolute improvements (Δ*R*^2^ > 0.15; error reductions >25% across metrics), as previously noted, but the relative uplifts in CNN-LSTM underscore the value of tailored feature integration for specific data completeness levels. Overall, while pollutant-based configurations offered negligible benefits, meteorological factors universally enhanced model efficacy, with the magnitude varying by architecture: hybrids such as CNN-LSTM and Transformer-LSTM showed the greatest potential for scalable air quality forecasting.

These findings align with El Mghouchi et al. (2024), who similarly observed that pollutant-based auxiliary variables provide limited predictive value, whereas meteorological factors substantially improve PM_2_._5_ forecasting accuracy.^35^^,^^36^ This disparity may arise due to the following factors: ① Pollutant-based variables such as CO and NO_2_ exhibit high correlations with PM_2_._5_ (exceeding 0.7), likely reflecting shared sources rather than direct causality, while the correlation between O_3_ and SO_2_ with PM_2_._5_ (ranging from 0.2 to 0.3) is lower, suggesting weaker source commonality. ② Table 3 shows that CO and NO_2_ are highly correlated, suggesting multicollinearity, but CO + NO_2_ slightly outperforms CO-only in most cases, indicating NO_2_ provides some unique information. It is probable that the deep-learning models’ robustness mitigates the impact of multicollinearity as much as possible, maintaining model stability and enhancing accuracy. ③ Meteorological factors (temperature, pressure, precipitation, wind speed), despite moderate correlations (0.2–0.4), exert a causal influence on PM_2_._5_ concentrations, actively contributing to their dilution or exacerbation through mechanisms such as dispersion or atmospheric stability.

**Table 3:** Distance correlation matrix

	CO	NO_2_	O_3_	SO_2_	PM_2.5_	T	P	RH	Prec	WS
CO	1.000	0.747	0.251	0.275	0.788	0.382	0.273	0.154	0.229	0.393
NO_2_	0.747	1.000	0.283	0.242	0.727	0.455	0.404	0.100	0.350	0.471
O_3_	0.251	0.283	1.000	0.446	0.294	0.734	0.638	0.544	0.096	0.206
SO_2_	0.275	0.242	0.446	1.000	0.205	0.277	0.197	0.339	0.114	0.086
PM_2.5_	0.788	0.727	0.294	0.205	1.000	0.491	0.331	0.127	0.257	0.340
T	0.382	0.455	0.734	0.277	0.491	1.000	0.811	0.195	0.256	0.290
P	0.273	0.404	0.638	0.197	0.331	0.811	1.000	0.148	0.290	0.323
RH	0.154	0.100	0.544	0.339	0.127	0.195	0.148	1.000	0.305	0.154
Prec	0.229	0.350	0.096	0.114	0.257	0.256	0.290	0.305	1.000	0.360
WS	0.393	0.471	0.206	0.086	0.340	0.290	0.323	0.154	0.360	1.000

T: temperature; P: pressure; RH: relative humidity; Prec: precipitation; WS: wind speed.

### Performance analysis of deep-learning algorithms

This analysis evaluates the performance of deep-learning algorithms (LSTM, CNN-LSTM, Transformer, and Transformer-LSTM) for PM_2_._5_ forecasting using complete (Category 1) and incomplete (Category 2) training data. For Category 1, considering that demonstration that cases with a training-to-prediction gap exceeding three months were excluded due to limited utility in Section “sensitivity for time gap between training and predicting period,” the analysis focuses on predictions immediately following the training period: long-term (Table S1), mid-term first six months (Table S2, Case 1), short-to-medium-term first quarter (Table S3, Case 1), and short-term first three months (Table S4, Cases 1–3). For Category 2, stable cases include Table S6, Case 1 (10 months training, 2 months prediction) and Table S5, Case 2 (9 months training, 3 months prediction), excluding anomalous cases (Table S6, Case 2; Table S5, Case 1). Performance is assessed using *R*^2^, MAE%, and RMSE%.

Transformer-LSTM, in both univariate and multivariate configurations, emerged as the most reliable algorithm across the eight cases spanning both categories, consistently demonstrating superior robustness and accuracy across all forecasting horizons. In the six cases of Category 1, it achieved higher prediction accuracy, while in the two cases of Category 2, it maintained reliability despite challenging conditions. In contrast, Transformer and CNN-LSTM followed with variable performance, struggling in certain instances, whereas LSTM consistently lagged, often producing negative outputs. This highlights Transformer-LSTM’s resilience, particularly in scenarios where other models, notably LSTM, underperformed.

### Insights into attention weights of Transformer-long short-term memory

In the multivariate configurations, we analyzed the Transformer-LSTM models, identified as the optimal deep learning algorithm among the four, by examining the attention weights allocated to different auxiliary variables, as derived from attention weight data extracted during the training process of our models.

In the meteorological combination, the attention weights revealed the following order of importance: temperature with the highest weight of approximately 0.2654, followed by pressure at 0.247, wind speed at 0.2055, and precipitation with the lowest weight of 0.0853. This ranking suggests that temperature plays the most dominant role in capturing the dynamic seasonal and diurnal influences on PM_2_._5_, while pressure and wind speed contribute moderately to atmospheric stability and dispersion effects, and precipitation has the least influence, likely due to its episodic nature.

In the O_3_ and SO_2_ combination, the attention weights indicated that SO_2_ received the highest weight of 0.42176, followed by O_3_ at 0.31576, implying that SO_2_ has a stronger association with PM_2_._5_ dynamics, potentially reflecting its role in local pollution sources, whereas O_3_’s contribution is notable but secondary, possibly linked to secondary aerosol formation. These attention weight distributions highlight the varying significance of input features across different configurations, with temperature and SO_2_ emerging as key drivers based on their respective weight rankings.

For the CO and NO_2_ combination, CO has the highest estimated weight of 0.4724, followed by NO_2_ with an estimated weight of 0.2546. This prioritization of NO_2_ over CO reflects its greater influence in short-term dynamics due to its direct involvement in nitrate formation from traffic emissions, overriding the stronger statistical association of CO. Additionally, for the analysis of attention weights across training time steps, we take one year of training data with a 7-day time window, for example, resulting in 359-time sequences. Among these, the weights for the initial approximately 60 sequences exhibited significant fluctuations, whereas the weights for the subsequent approximately 300 sequences remained relatively uniform with minimal fluctuations and overall higher weights. The initial instability in attention weights, likely due to model warm-up, seasonal transitions, and limited historical context, underscores the importance of a stabilization period to adapt to evolving PM_2_._5_ patterns. The subsequent stability and higher weights, reflecting the convergence and recognition of seasonal cycles, highlight the model’s reliance on complete temporal data for robust performance.

### Multi-sites analysis and verification

To improve the generalizability of our findings from the previous six subsections, we performed an extended validation of the optimal Transformer-LSTM model incorporating meteorological factors. We trained the model using complete four-season data to predict air quality for the following periods: one year (November 2023–October 2024), the first half-year (November 2023–April 2024), and the first quarter (November 2023–January 2024). This validation was conducted in two heavily polluted Chinese cities, Urumqi and Hangzhou, which frequently rank among the top 10% of Chinese cities for PM2.5 pollution. The results show stable model performance across both cities, with *R*^2^ values ranging from 0.539 to 0.627, MAE% from 28.8% to 36%, and RMSE% from 46.8% to 56.3% (see Table S7).

This multi-site validation highlights the meteorological-based Transformer-LSTM model’s potential for air quality management in heavily polluted cities, with stable yearly, half-yearly, and quarterly forecasts ensuring timely predictions.

### Key findings

The key findings are.

(1) **Transformer-LSTM Outperforms**: Transformer-LSTM surpassed LSTM, CNN-LSTM, and Transformer, with higher *R*^2^ and lower MAE% and RMSE% in univariate and multivariate setups, excelling in monthly, seasonal, and annual PM_2.5_ forecasts due to its self-attention and sequential modeling.
(2) **Meteorological Factors Boost Accuracy**: Meteorological factors (temperature, pressure, precipitation, wind speed) significantly enhanced prediction accuracy over univariate models, reflecting their causal role in PM_2.5_ dynamics.
(3) **Pollutant Configurations Underperform**: Pollutant-based configurations (CO + NO_2_, CO-only, O_3_+SO_2_) showed minimal predictive improvement, indicating shared emission sources rather than direct causality.
(4) **Complete Data Enhances Performance**: Models trained on complete four-season data (Category 1) outperformed those with incomplete seasonal data (Category 2), highlighting the need for full seasonal patterns to ensure robust forecasting.
(5) **Temporal Gap Sensitivity**: Prediction accuracy declined with temporal gaps exceeding three months between training and prediction periods, as training data became less representative of evolving PM_2.5_ dynamics, such as seasonal shifts or new pollution sources.
(6) **Training Proportion Impact:** For incomplete datasets, a higher training data proportion generally improved performance, but single-month forecasts exhibited diminishing returns, likely due to limited temporal windows for capturing trends.

### Policy implications

The Transformer-LSTM model’s consistent stability across different forecasting horizons underscores its reliability as a tool for policymakers in cities like Chengdu to effectively predict and manage air pollution. There is a need for policies that incorporate real-time meteorological data into air quality monitoring systems, enabling more accurate and timely responses. Moreover, training models with complete seasonal datasets and avoiding temporal gaps longer than three months is crucial to ensure forecasting accuracy. Policymakers should prioritize the use of comprehensive and up-to-date datasets in air quality prediction systems to better capture the evolving dynamics of PM_2.5_ concentrations. Such data-driven approaches can support evidence-based strategies, including targeted pollution control measures and urban planning initiatives aimed at reducing PM_2.5_ levels.

### Limitations of this study

The study’s reliance on Chengdu data limits generalizability, as PM_2.5_ dynamics vary across regions with different topographies, emission profiles, and climates, such as coastal cities with strong sea breezes, arid regions with dust contributions, or rural areas with biomass burning. The dataset, spanning only two years (November 2022–October 2024), restricts the model’s ability to capture long-term PM_2.5_ trends influenced by decadal climatic shifts or policy changes.

Future research should: (1) validate the Transformer-LSTM model across diverse regions, including provinces, cities, and countries with varied environmental conditions (e.g., coastal Shanghai, arid Lanzhou, or tropical Hainan), to ensure robust generalizability and (2) incorporate longer datasets, spanning a decade or more, to enhance the model’s ability to predict long-term PM_2.5_ trends and account for multi-year variations in emissions and climate.

## Resource availability

### Lead contact

Further information and requests for resources and reagents should be directed to and will be fulfilled by the lead contact, Ling Zeng (zengling18@cdut.edu.cn).

### Materials availability

As this study did not generate new unique material, the material availability is not applicable.

### Data and code availability

• Data are available online at http://eia-data.com/ or https://doi.org/10.5281/zenodo.18229490.
• Any code request will be made through the lead contact.
• Any additional information required to reanalyze the data reported in this article is available from the lead contact upon request.

## Acknowledgments

This study was supported by the National Science and Technology Major Project for Deep Earth (No. 2025ZD1008103) and the Deep Earth Probe and Mineral Resources Exploration - National Science and Technology Major Project (No. 2024ZD1001200). We also thank Bin Hu for his support in revising and validating this article.

## Author contributions

Ling Zeng: writing – original draft, writing – review and editing, validation, methodology, and conceptualization. Runan Dong: resources, data curation, formal analysis, and visualization. Meng Yuan: visualization, formal analysis, and investigation. Linhai Jing: formal analysis, validation, fund acquisition, investigation, and supervision. Shoutao Jiao: investigation.

## Declaration of interests

The authors declare no competing interests.

## STAR★Methods

### Key resources table

**Table:** 

REAGENT or RESOURCE	SOURCE	IDENTIFIER
**Deposited data**		

Data is available online	Environmental Meteorological Data Service Platform	http://eia-data.com/orhttps://doi.org/10.5281/zenodo.18229490

**Software and algorithms**		

Software — MATLAB r2024a	MathWorks	https://www.mathworks.com/products/new_products/release2024a.html
Deep Learning Toolbox (for LSTM, CNN-LSTM, Transformer, and Transformer-LSTM models)	MathWorks	https://www.mathworks.com/products/deep-learning.html

### Experimental model and study participant details

This study does not involve experimental models or study participants typical in the life sciences.

### Method details

#### Data collection

**Figure 1:**
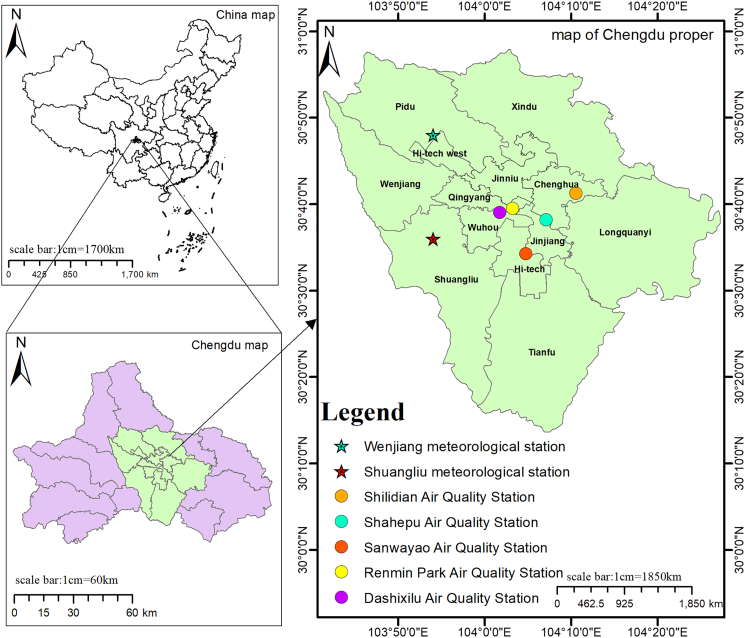
The location of the study area The top left shows a map of China, scale bars: 1 cm = 1700 km. The bottom left shows a map of Chengdu, scale bars: 1 cm = 60 km, and the right side shows a map of Chengdu’s main urban area, scale bars: 1 cm = 1850 km.

The study area is located at Chengdu, Sichuan Province (Figure 1), encompassing five air quality monitoring stations and two meteorological monitoring stations that provide air quality and meteorological data, respectively. The five air quality monitoring stations consist of Shilidian station (1432A), Shahepu station (1434A), Renmin Park station (1437A), Dashi West station (2880A), Sanwayao station (1433A). Datasets for NO_2_, CO, SO_2_, O_3_, and PM_2.5_ are collected from the five air quality stations mentioned above, ranging from November 1^st^ 2022 to October 31^th^ 2024. The two meteorological stations, Wenjiang station and Shuangliu station, provided datasets of temperature (T), pressure (P), relative humidity (RH), precipitation (Prec) and wind speed (WS) are collected over the same period. Average values from five air quality stations and the two metrological stations were used to represent the overall spatial quality and meteorological data for the study area, respectively.

#### Data preprocessing and exploratory analyses

##### Data cleaning

Here preprocessing mainly consist of outliers’ removal and data standardization. Outliers were detected based on the analysis of box-and-whisker plot and no actual outliers were detected.^37^^,^^38^ And next, datasets are standardized to eliminate the impact of dimensions, using the min-max scaling method as following equation:

(Equation 1) $$X_{i,standardized}=\frac{X_{i}-X_{\min}}{X_{\max}-X_{\min}}$$

The entire preprocessing process was executed using MATLAB programming. Additionally, there were no missing data in the datasets, so no treatment for missing value was required.

##### Distance correlation analysis

Distance correlation (*dCor*), introduced by Székely et al.,^39^ is a robust statistical method to measure both linear and nonlinear dependencies between two random variables *X*∈*R* and *Y*∈*R*. Unlike Pearson’s correlation, which captures only linear relationships, distance correlation leverages Euclidean distance matrices to detect any form of dependence, with a value of zero indicating independence (assuming finite first moments). It computes the distance covariance *dCor*^2^(*X*,*Y*) as the mean product of centered distance matrices:

$$A_{ij}=\left\|x_{i}-x_{j}\right\|-{\bar{a}}_{i.}-{\bar{a}}_{.j}+{\bar{a}}_{..}$$

$$B_{ij}=\left\|y_{i}-y_{j}\right\|-{\bar{b}}_{i.}-{\bar{b}}_{.j}+{\bar{b}}_{..}$$

$$d{Cor}^{2}\left(X,Y\right)=\frac{1}{n^{2}}\sum_{i=1}^{n}\sum_{j=1}^{n}A_{ij}\times B_{ij}$$

And then normalizes it to obtain the distance correlation coefficient as follow:

(Equation 2) $$R^{2}\left(X,Y\right)=\frac{d{Cor}^{2}\left(X,Y\right)}{\sqrt{d{Cor}^{2}\left(X,X\right)d{Cor}^{2}\left(Y,Y\right)}}$$

Based on the distance correlation analysis, relative humidity (RH) was excluded as a potential auxiliary variable for PM_2.5_ prediction due to its low correlation with PM_2.5_ (0.127), falling even below 0.2, indicating minimal predictive value. The remaining eight variables—CO (0.788), NO_2_ (0.727), T (0.491), WS (0.340), P (0.331), O_3_ (0.294), Prec (0.257), and SO_2_ (0.205)—were retained as potential predictors due to their stronger correlations with PM_2.5_. However, significant intercorrelations were observed, notably CO-NO_2_ (0.747) and T-P (0.811), alongside O_3_-T (0.734) and O_3_-P (0.638), with a moderate correlation between NO_2_-T (0.455), suggesting shared emission sources or meteorological influences that may introduce redundancy. This distance correlation analysis serves as a preliminary screening to identify promising predictors, with subsequent analysis planned to rigorously confirm each variable’s predictive significance for accurate PM_2.5_ prediction.

##### Descriptive statistics

We statistically analyzed five pollutant concentrations (CO, NO_2_, O_3_, SO_2_, PM_2_._5_) and four meteorological factors (temperature [T], pressure [P], precipitation [Prec], and wind speed [WS]) across two study periods in Chengdu: November 1, 2022–October 31, 2023, and November 1, 2023–October 31, 2024. Mean values represent average concentrations, variances quantify data spead, and the coefficient of variation (CV, standard deviation/mean) measures relative variability, enabling comparisons across pollutants and meteorological variables.

Below Table summarizes the results. Mean concentrations of NO_2_ and PM_2_._5_ decreased, while CO, O_3_, and SO_2_ increased from the first to the second period. Variances increased for CO and PM_2_._5_, indicating greater fluctuations, but decreased for NO_2_, O_3_, and SO_2_, suggesting more stable concentrations. CVs rose for CO, NO_2_, and PM_2_._5_, reflecting higher relative variability, while CVs for O_3_ and SO_2_ fell, indicating consistency. Meteorological factors showed minimal changes with limited impact on air quality trends.

**Table:** Statistical analyses of CO, NO_2_, and PM_2.5_ concentrations in Chengdu

Descriptive statistic	Mean values		Variances		Coefficients of variation (CV)	
**Period**	2022.11.01–2023.10.31	2023.11.01–2024.10.31	2022.11.01–2023.10.31	2023.11.01–2024.10.31	2022.11.01–2023.10.31	2023.11.01–2024.10.31
CO	0.614027	0.633115	0.026585	0.038631	0.265541	0.310445
NO_2_	30.85479	28.33005	174.7649	163.2194	0.428454	0.450961
PM_2.5_	39.44822	36.54536	708.0643	836.58	0.674542	0.791446
O_3_	95.74904	102.1415	2599.497	2486.815	0.532489	0.488224
SO_2_	3.116712	3.380328	1.115242	0.915557	0.338835	0.283064
T	18.1417	18.53802	56.23795	65.0431	0.413368	0.435048
P	950.9589	950.6199	56.89198	62.96936	0.007932	0.008348
Prec	1.472852	1.46744	23.34876	19.3945	3.280747	3.00109
WS	1.708518	1.703272	0.346479	0.332324	0.344523	0.338452

##### Descriptive seasonal trends

**Figure 2:**
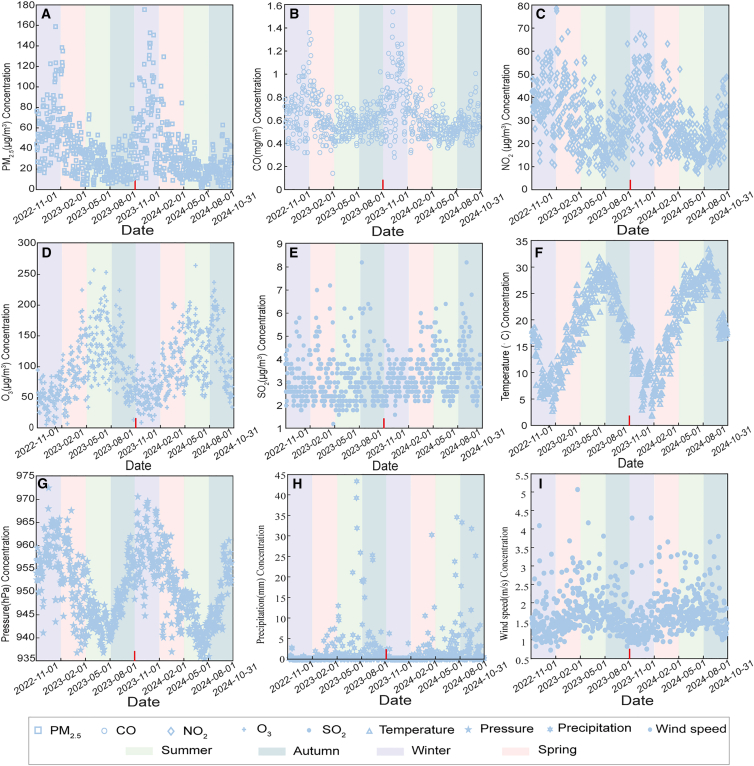
The trends of pollutants (NO_2_, CO, PM_2_._5_, O_3_, and SO_2_) and meteorological factors (temperature, pressure, precipitation, and wind speed) in Chengdu from November 2022 to October 2024 (A) PM_2_._5_ concentrations. (B) CO concentrations. (C) NO_2_ concentrations. (D) O_3_ concentrations. (E) SO_2_ concentrations. (F) Temperature. (G) Pressure. (H) Precipitation. (I) Wind speed.

The trends of five pollutants and four meteorological factors are described in the following Figure 2, covering November 2022 to October 2024 in Chengdu. PM_2_._5_, CO, and NO_2_ exhibit clear seasonal trends, with concentrations peaking in winter and decreasing in summer, likely due to heating activities, temperature inversions, and regional pollution events. Conversely, O_3_ shows a distinct pattern, with higher concentrations in summer and lower in winter, driven by increased photochemical activity during warmer months. SO_2_ trends are less pronounced, lacking a clear seasonal pattern, possibly due to more consistent emission sources.

Among meteorological factors, pressure closely follows temperature trends with slight seasonal fluctuations. Precipitation displays anomalously high values during a small portion of summer, likely aiding pollutant dispersion. Wind speed is slightly higher on average in spring and autumn, but its seasonal fluctuations are not pronounced, with occasional anomalously high values. These patterns highlight the influence of seasonal and meteorological factors on air quality dynamics.

#### Time series analyses methods

##### Long short-term memory (LSTM)

**Figure 3:**
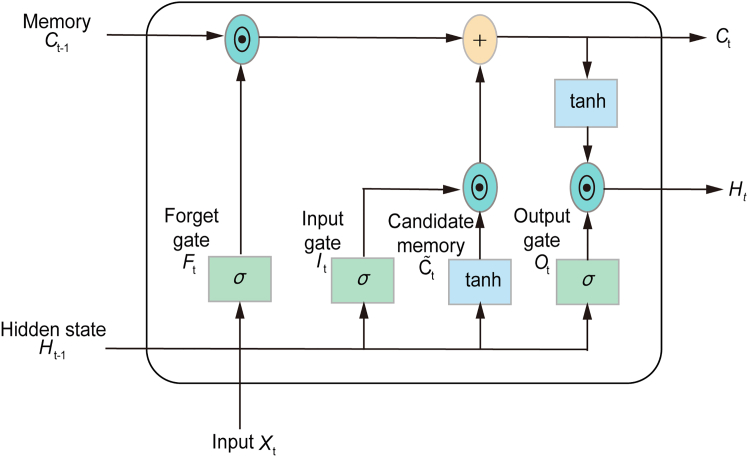
The architecture of LSTM This diagram illustrates the internal architecture and data flow, highlighting the three gating mechanisms (forget, input, output) that regulate the update of the cell state (Ct) and the generation of the hidden state (Ht).

Long Short-Term Memory (LSTM), a specialized recurrent neural network (RNN), addresses traditional RNNs’ limitations in modeling long-term dependencies in sequential data.^25^ Its core strength lies in a memory cell that stores and selectively updates information over extended periods, ideal for time series tasks like PM_2_._5_ prediction.^40^ Three gates regulate this cell: the forget gate discards irrelevant past data, the input gate incorporates new relevant information, and the output gate controls what advances to the next step. The LSTM architecture is depicted in Figure 3. LSTM effectively captures both short- and long-term dependencies in time series data. However, it struggles with extremely long-term dependencies and remains sensitive to data fluctuations.

##### CNN-LSTM

Convolutional Neural Networks (CNNs) excel at extracting local features, typically in image processing, but their convolutional operations—using filters to detect trends, peaks, and variations—also apply to time series. Pooling layers then reduce dimensionality, boosting efficiency.

**Figure 4:**
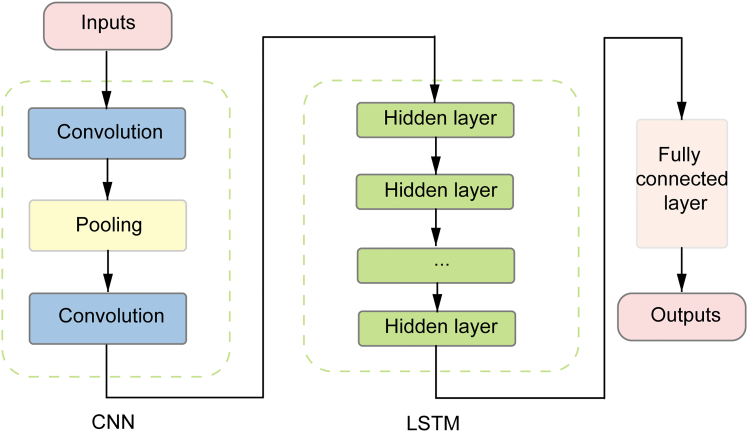
The architecture of CNN-LSTM The model sequentially integrates a convolutional neural network (CNN) and a long short-term memory (LSTM) network for temporal pattern capture, with a fully connected layer for final output generation.

The CNN-LSTM hybrid integrates CNN’s local pattern extraction with LSTM’s long-term dependency modeling for time series analysis.^41^ Its architecture (Figure 4) features a CNN applying 1D convolution to capture short-term trends and joint features from inputs (e.g., CO, SO_2_, NO_2_, O_3_), followed by an LSTM that processes these features to model temporal dependencies. This hybrid model enhances PM_2_._5_ predictive accuracy by utilizing CNN to extract meaningful local features from the time series, thereby reducing input complexity, and allowing LSTM to effectively capture temporal dependencies.^24^

##### Transformer

**Figure 5:**
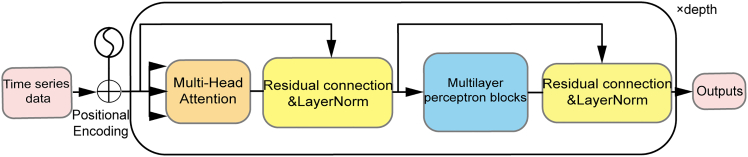
The architecture of the Transformer The diagram illustrates the flow of sequential data through positional encoding, multi-head attention mechanisms, and feedforward networks with residual connections and layer normalization.

The Transformer, introduced by Vaswani et al. (2017), is a deep learning architecture that transforms sequence modeling by replacing sequential processing (as in RNNs and LSTMs) with a self-attention mechanism.^30^ This enables parallel input processing, enhancing training efficiency and capturing long-term dependencies effectively. Self-attention weights input elements based on their mutual relationships, excelling at contextual analysis in time series data. Its architecture (Figure 5) comprises encoder and decoder blocks with multi-head self-attention and feedforward layers, using positional encoding to maintain sequence order.

Transformers outperform LSTMs in tasks like air quality, financial, and energy forecasting, with superior long-range dependency modeling and faster training. However, they face higher computational complexity and reduced efficiency with very long sequences due to attention dilution.

##### Transformer-LSTM

**Figure 6:**
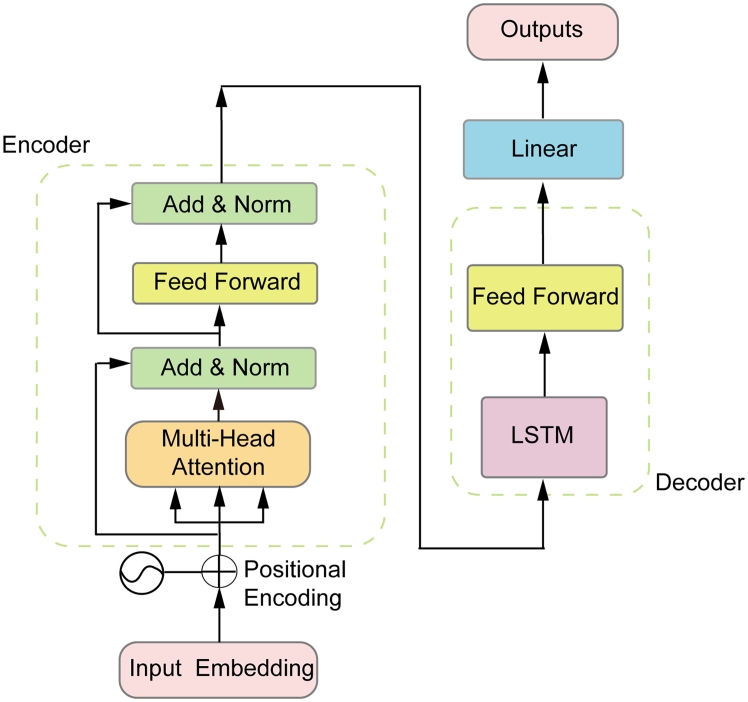
The architecture of the Transformer-LSTM Featuring an encoder-decoder structure that processes input via embedding, multi-head attention mechanisms, and feedforward layers, with the decoder incorporating an LSTM for enhanced sequential output generation.

The Transformer-LSTM model integrates the Transformer’s parallel processing and self-attention with LSTM’s sequential, memory-based modeling. In this hybrid structure, the Transformer extracts key temporal features via self-attention, feeding them into the LSTM layer to refine time series forecasting by capturing sequential dependencies and long-term memory. Illustrated in Figure 6, this architecture shines in air pollution forecasting, stock price prediction, and meteorological analysis, utilizing strong feature extraction and sequence retention. However, it inherits drawbacks from both models: high computational complexity, challenges in hyperparameter tuning, and a risk of overfitting on small datasets.

### Quantification and statistical analysis

Model performance is assessed using Mean Absolute Error (MAE), Root-Mean-Square Error (RMSE), R-squared (*R*^2^), and their percentage-based variants, MAE% and RMSE%. MAE measures the average magnitude of prediction errors, RMSE emphasizes larger errors, and *R*^2^quantifies the proportion of variance in actual PM_2_._5_ values explained by the model. MAE% and RMSE% normalize errors by the mean of actual values, enabling consistent comparisons across forecasting horizons with varying PM_2_._5_ scales.

#### Mean absolute error (MAE) and MAE%

MAE calculates the average absolute difference between actual and predicted values, offering a straightforward, outlier-insensitive error metric.^42^ It is defined as:

(Equation 3) $$MAE=\frac{1}{n}\sum_{i=1}^{n}\left|y_{i}-\hat{y_{i}}\right|$$

where *y*_*i*_ represents the actual values, $\hat{y_{i}}$ represents the predicted values, *n* is the total number of observations. Lower MAE reflects predictions closer to actual values, though it weighs all errors equally.

MAE% normalizes MAE by the mean of actual values, expressed as percentages, to account for varying PM_2_._5_ scales across scenarios. It is defined as:

(Equation 4) $$MAE\%=100\times MAE/\bar{y}$$

where $\bar{y}=\left(1/n\right)\sum_{i=1}^{n}y_{i}$ is the mean of actual values.

#### Root means square error (RMSE) and RMSE%

RMSE measures prediction error by averaging squared differences between actual and predicted values, then taking the square root. It penalizes larger errors more than MAE, making it ideal when big deviations matter.^42^ It is defined as

(Equation 5) $$RMSE=\sqrt{\frac{1}{n}\sum_{i=1}^{n}{\left(y_{i}-\hat{y_{i}}\right)}^{2}}$$

where *y*_*i*_, $\hat{y_{i}}$, and *n* are those as mentioned above.

RMSE% normalizes RMSE by the mean of actual values, expressed as percentages, to account for varying PM_2_._5_ scales across scenarios. It is defined as:

(Equation 6) $$RMSE\%=100\times RMSE/\bar{y}$$

where ȳ is the mean of actual values, as defined above.

#### R-squared (***R***^2^)

*R*^2^ is unitless and describes the proportion of variance in the dependent variable that is explained by the independent variables.^43^ It shows how well the regression model explains the variability of the data and provides a unitless measure of model fit. It is defined as:

(Equation 7) $$R^{2}=1-\frac{\sum_{i=1}^{n}{\left(y_{i}-\hat{y_{i}}\right)}^{2}}{\sum_{i=1}^{n}{\left(y_{i}-\bar{y}\right)}^{2}}$$

where *y*_*i*_, $\hat{y_{i}}$, and *n* are those as mentioned above. $\bar{y}$ is the mean of the actual values. Ranging from 0 to 1, *R*^2^ = 1 indicates perfect variance explanation, while 0 suggests no improvement over the mean. Negative *R*^2^ can occur if the model underperforms the mean. Higher*R*^2^ values signify better fit.
