# Supplementary material for: Evaluating deep learning time series models for PM2.5 forecasting across diverse horizons
Source: iScience. 2026 Jan 21;29(2):114770. doi: 10.1016/j.isci.2026.114770 (PMC12907896; doi:10.1016/j.isci.2026.114770)
Supplement: Document S1. Figures S1–S23 and Tables S1–S7 [file mmc1.pdf]

**iScience, Volume *29***

## Supplemental information

# Evaluating deep learning time series models for $PM_{2.5}$ forecasting across diverse horizons

**Ling Zeng, Runan Dong, Meng Yuan, Linhai Jing, and Shoutao Jiao**

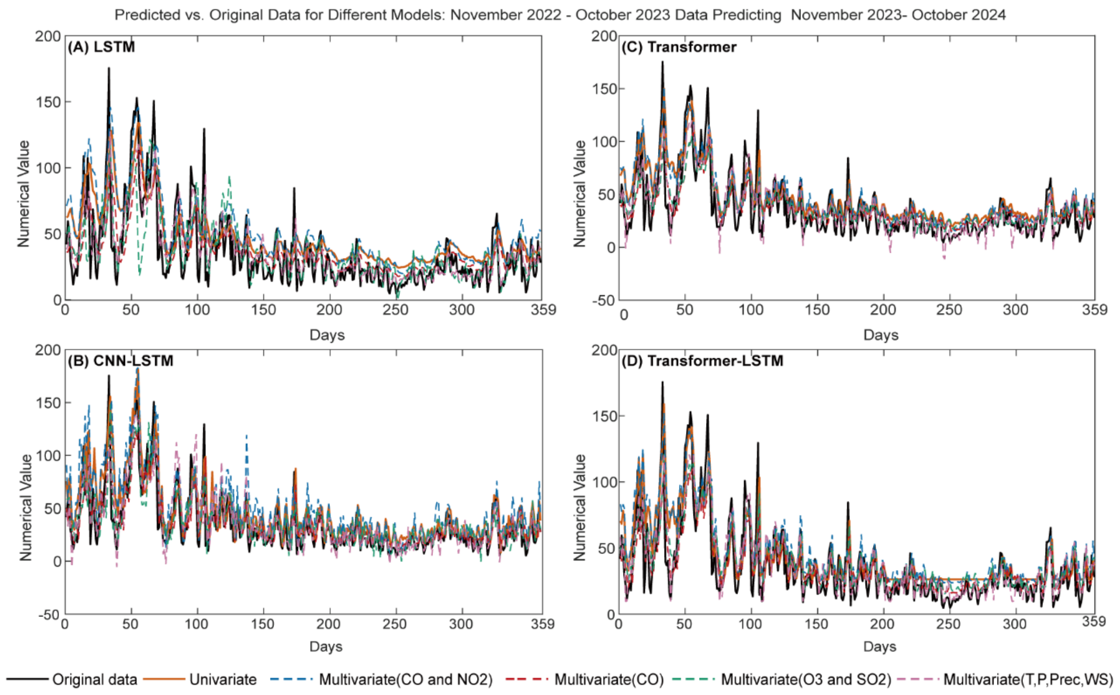


Fig. S1. Predicted vs. Original Data for Different deep-learning Models Category 1-1 Long-term predictions

(A)LSTM (B) CNN-LSTM (C) Transformer (D) Transformer-LSTM

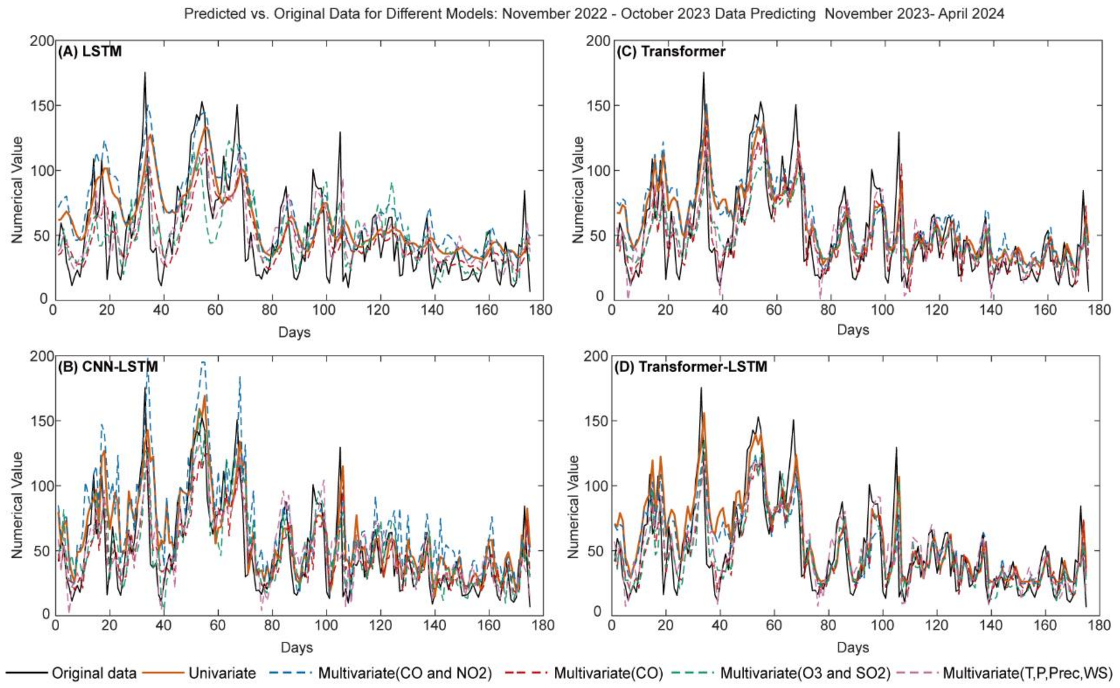


Fig. S2. Predicted vs. Original Data for Different Models Category1-2_Case 1 Mid-term predictions

(A)LSTM (B) CNN-LSTM (C) Transformer (D) Transformer-LSTM

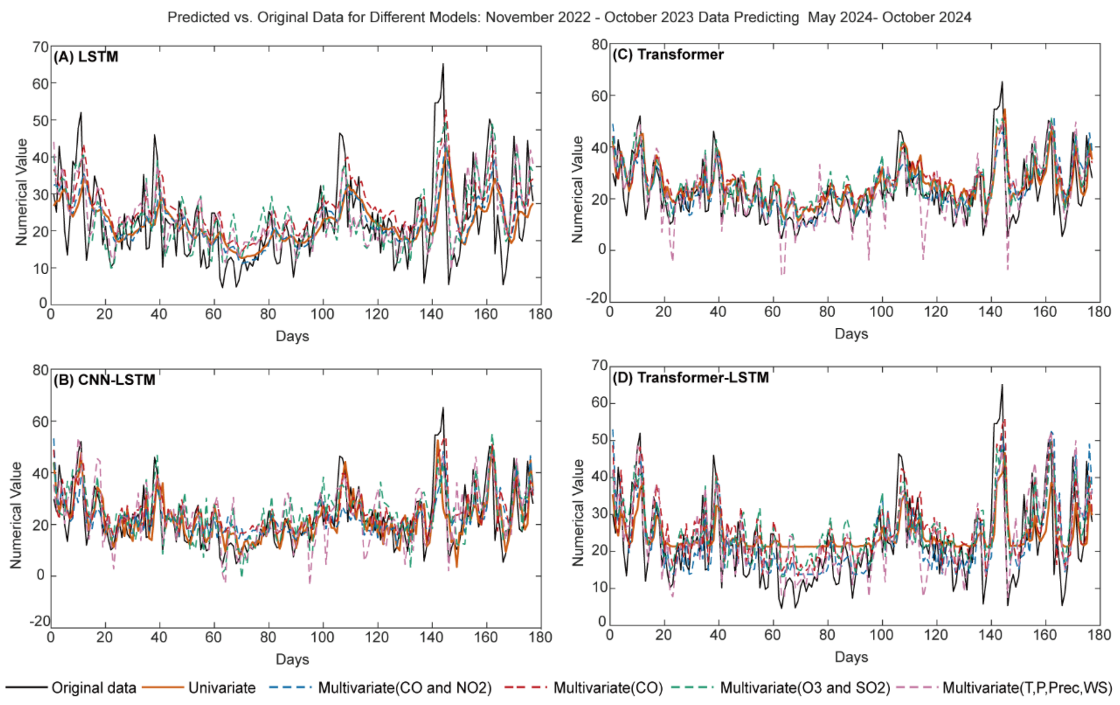


Fig. S3. Predicted vs. Original Data for Different Models Category1-2_Case 2 Mid-term predictions

(A)LSTM (B) CNN-LSTM (C) Transformer (D) Transformer-LSTM

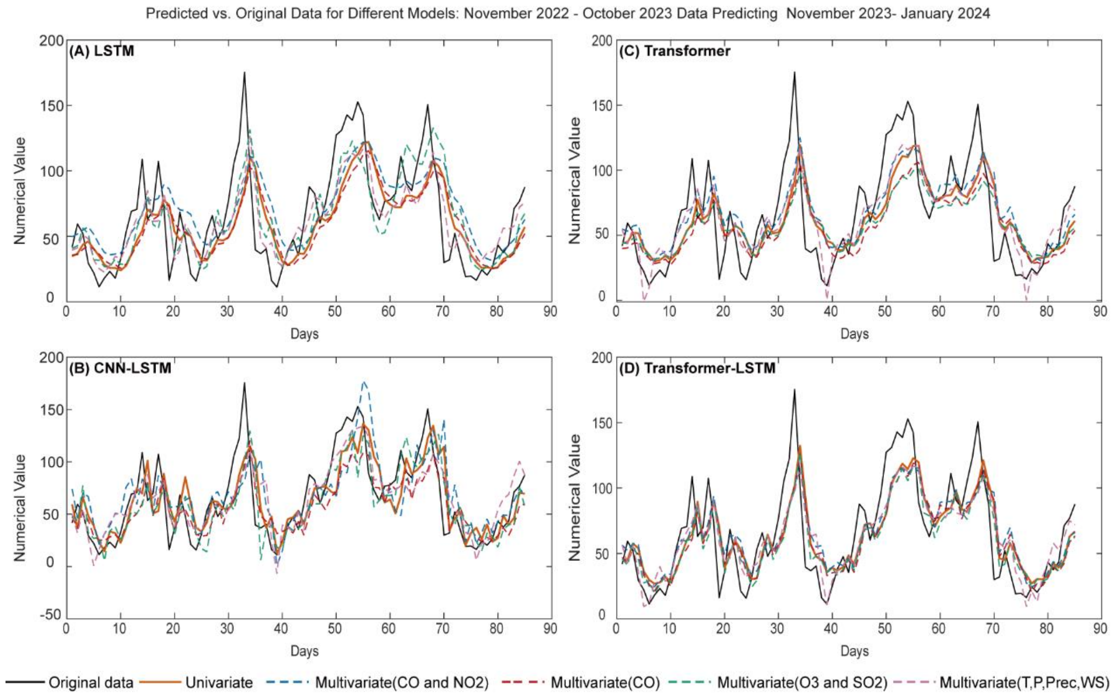


Fig. S4. Predicted vs. Original Data for Different Models Category1-3_Case 1 Short-to-medium-term seasonal predictions

(A)LSTM (B) CNN-LSTM (C) Transformer (D) Transformer-LSTM

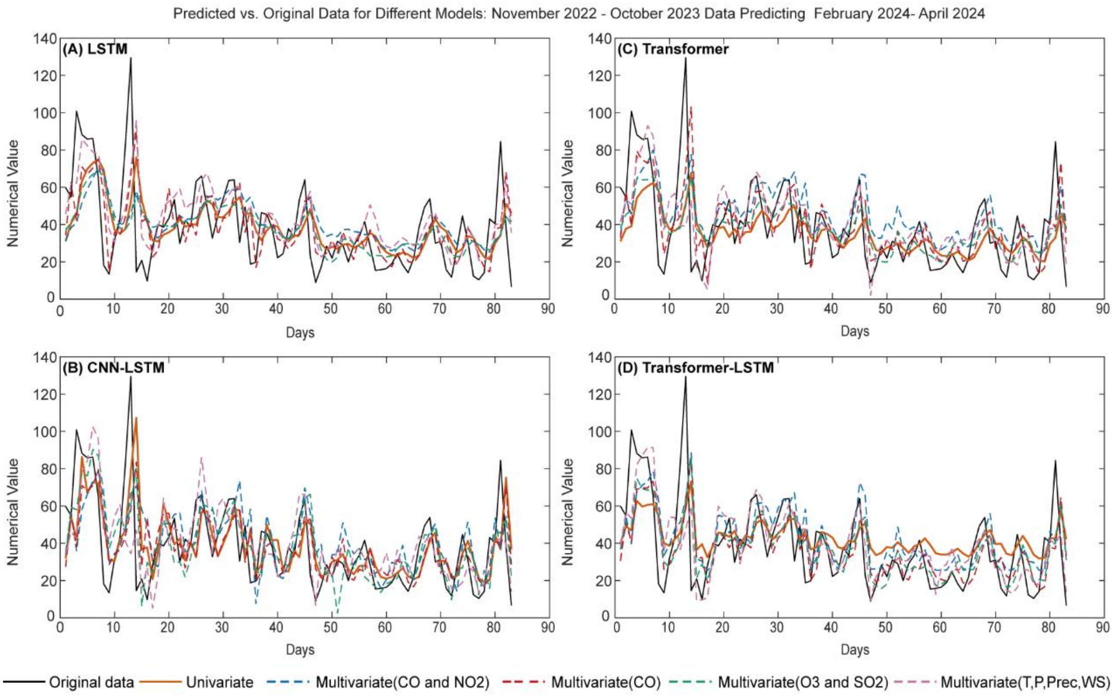


Fig. S5. Predicted vs. Original Data for Different Models Category1-3_Case 2 Short-to-medium-term seasonal predictions

(A)LSTM (B) CNN-LSTM (C) Transformer (D) Transformer-LSTM

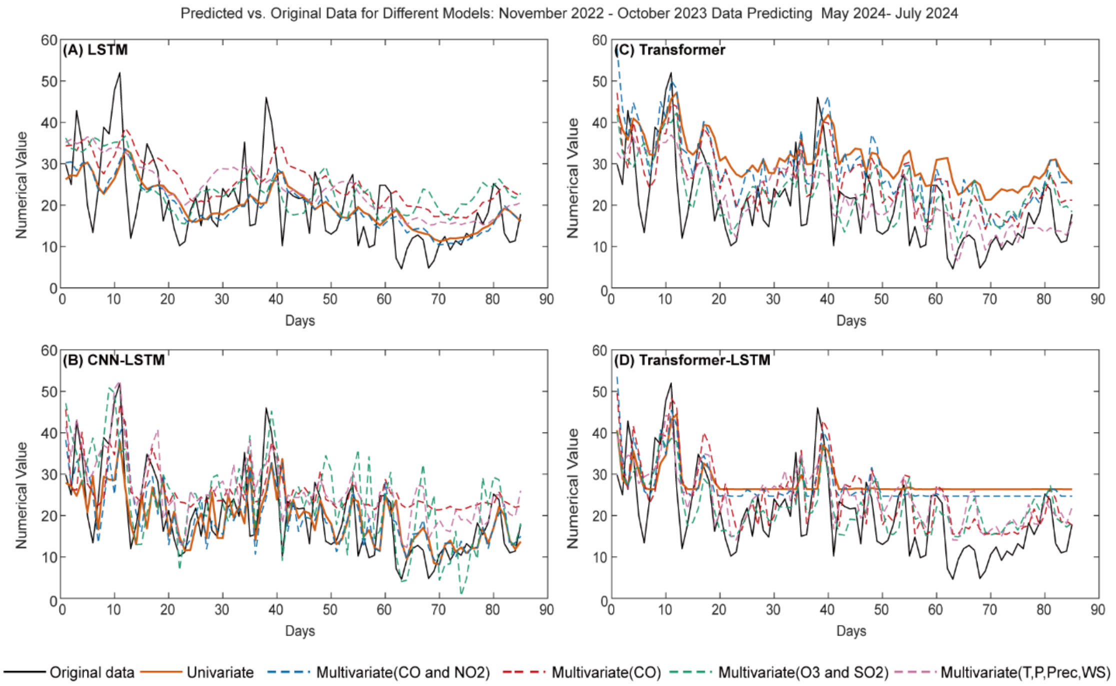


Fig. S6. Predicted vs. Original Data for Different Models Category1-3_Case 3 Short-to-medium-term seasonal predictions

(A)LSTM (B) CNN-LSTM (C) Transformer (D) Transformer-LSTM

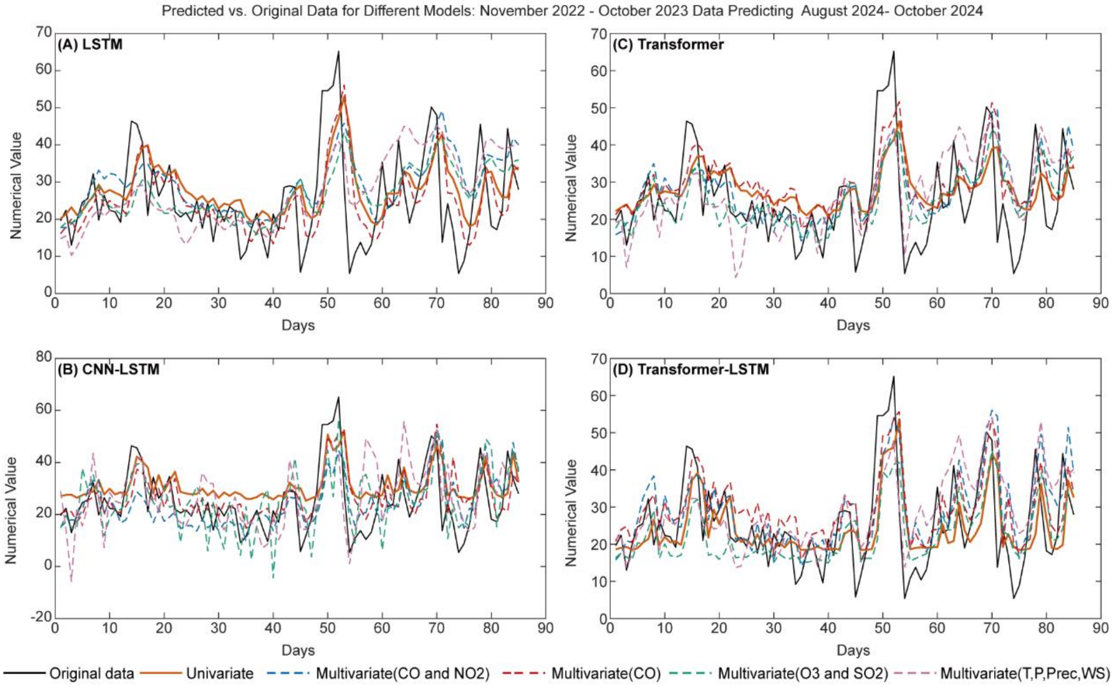


Fig. S7. Predicted vs. Original Data for Different Models Category1-3_Case 4 Short-to-medium-term seasonal predictions

(A)LSTM (B) CNN-LSTM (C) Transformer (D) Transformer-LSTM

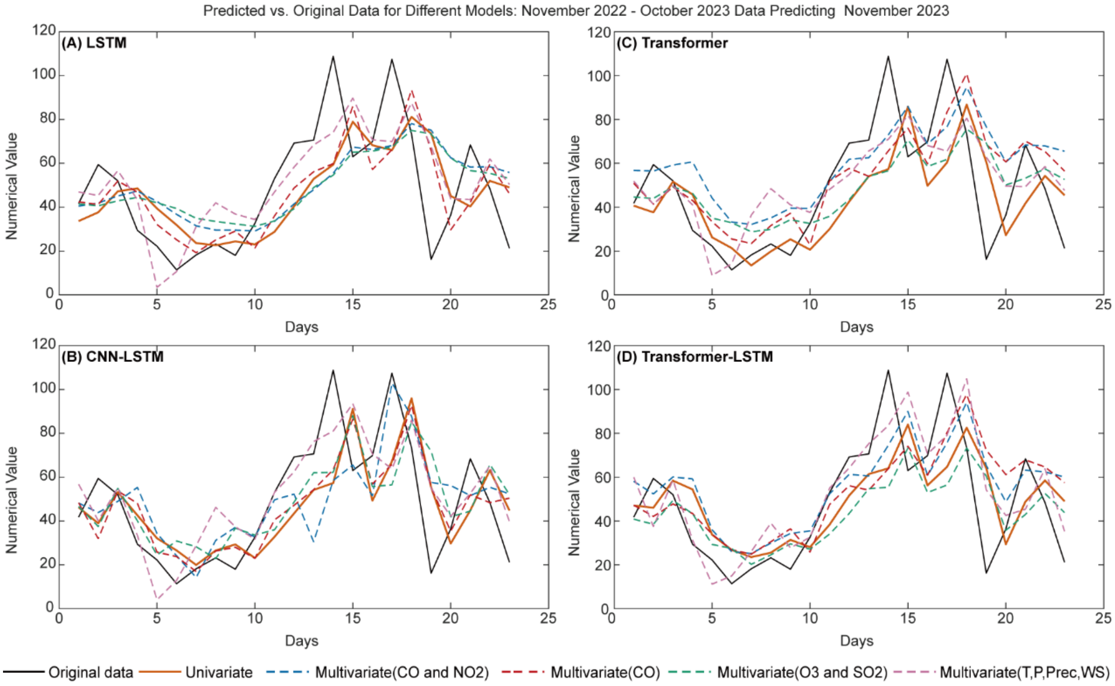


Fig. S8. Predicted vs. Original Data for Different Models Category1-4_Case 1 Short-

term monthly predictions

(A)LSTM (B) CNN-LSTM (C) Transformer (D) Transformer-LSTM

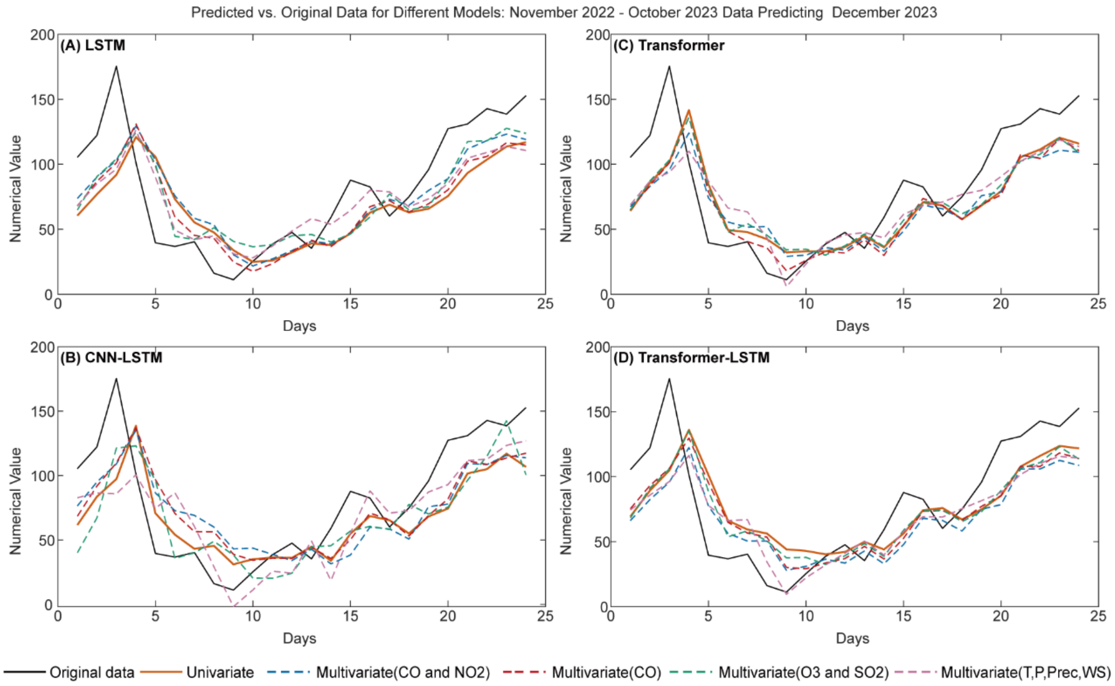


Fig. S9. Predicted vs. Original Data for Different Models Category1-4_Case 2 Short-term monthly predictions

(A)LSTM (B) CNN-LSTM (C) Transformer (D) Transformer-LSTM

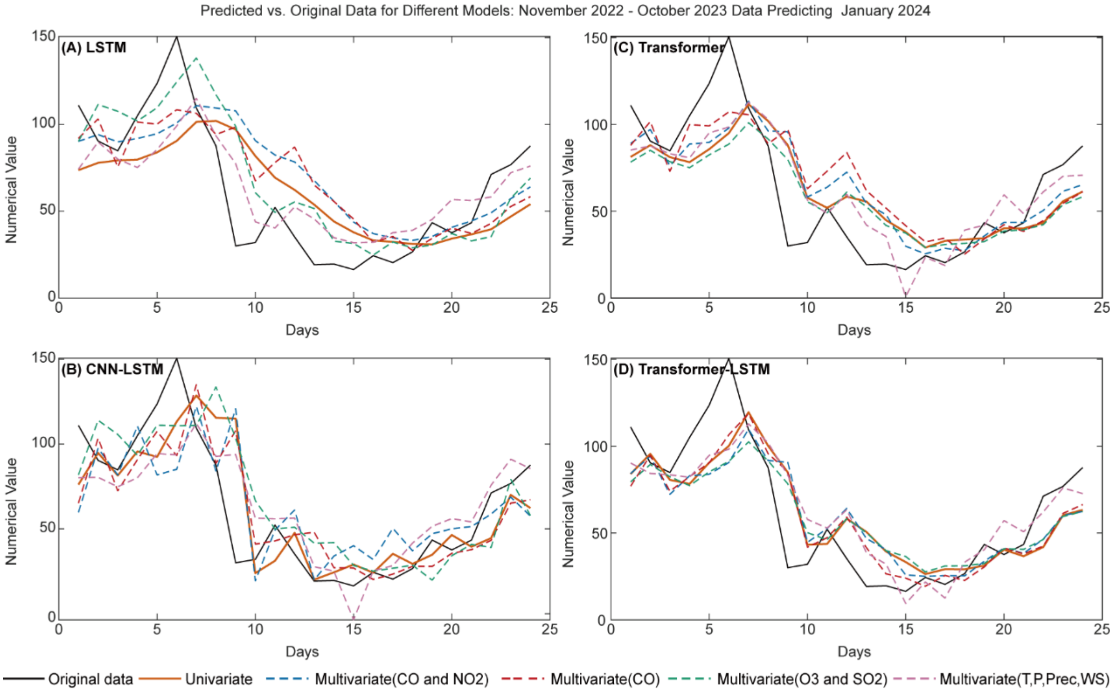

Fig. S10. Predicted vs. Original Data for Different Models Category1-4_Case 3 Short-term monthly predictions

(A)LSTM (B) CNN-LSTM (C) Transformer (D) Transformer-LSTM

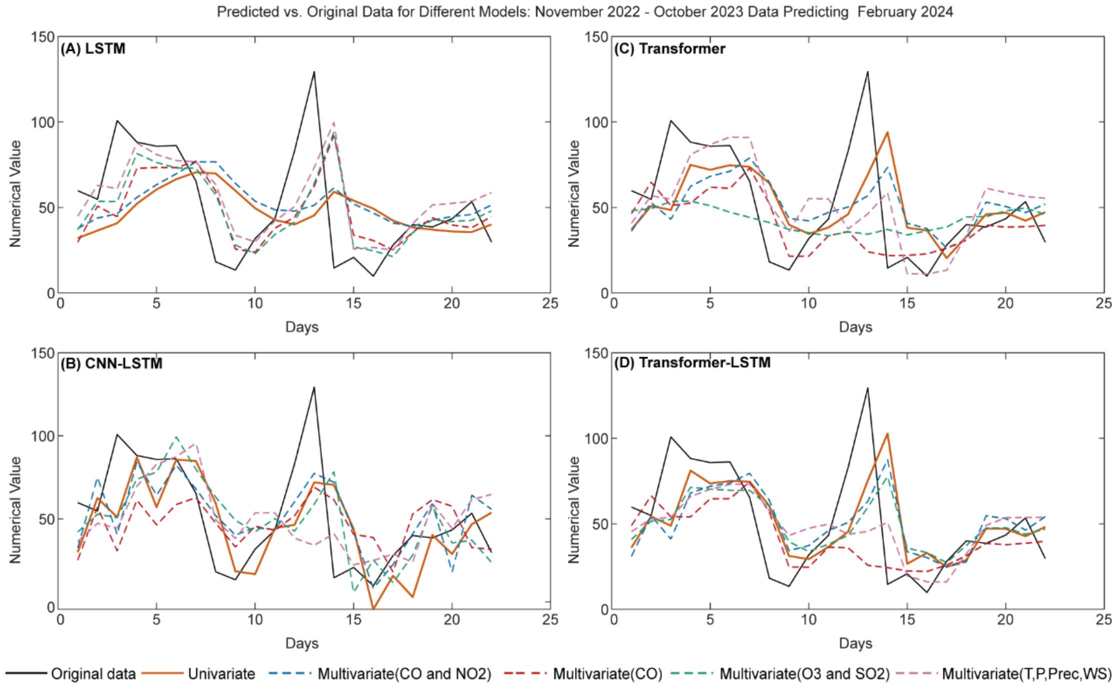


Fig. S11. Predicted vs. Original Data for Different Models Category1-4_Case 4 Short-term monthly predictions

(A)LSTM (B) CNN-LSTM (C) Transformer (D) Transformer-LSTM

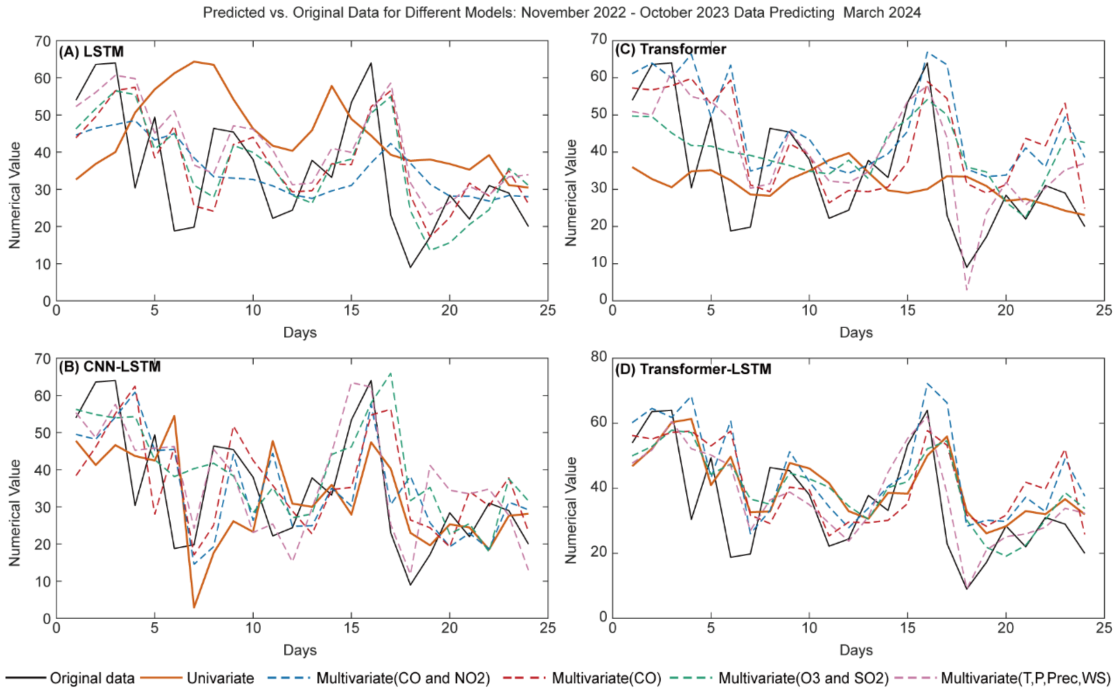


Fig. S12. Predicted vs. Original Data for Different Models Category1-4_Case 5 Short-term monthly predictions

(A)LSTM (B) CNN-LSTM (C) Transformer (D) Transformer-LSTM

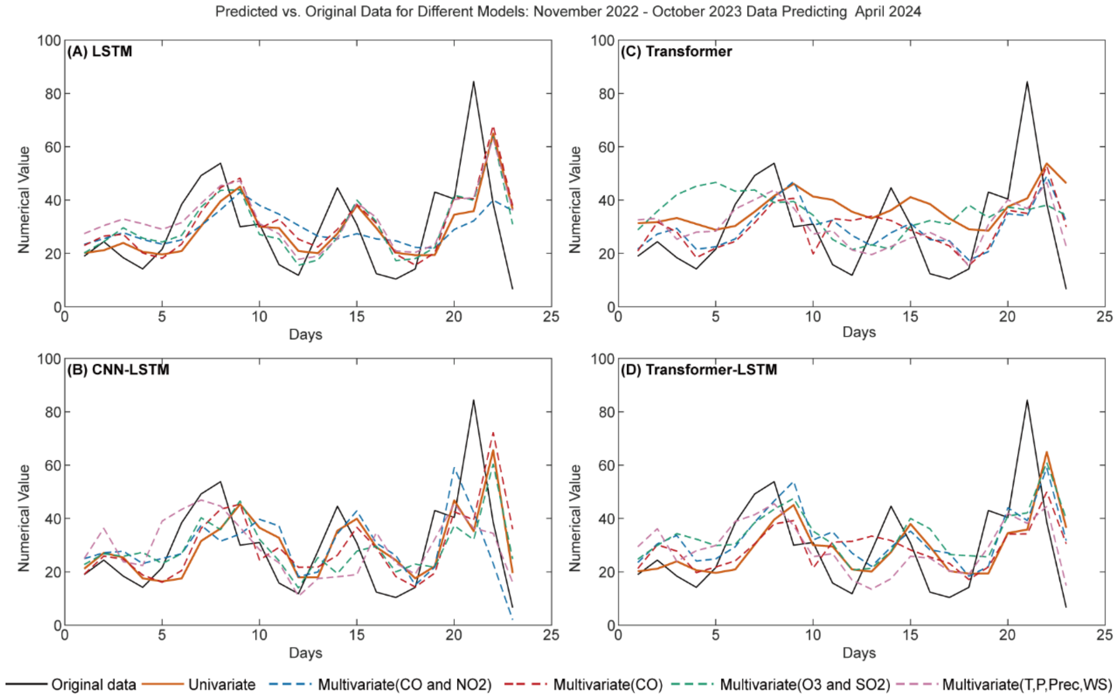


Fig. S13. Predicted vs. Original Data for Different Models Category1-4_Case 6 Short-term monthly predictions

(A)LSTM (B) CNN-LSTM (C) Transformer (D) Transformer-LSTM

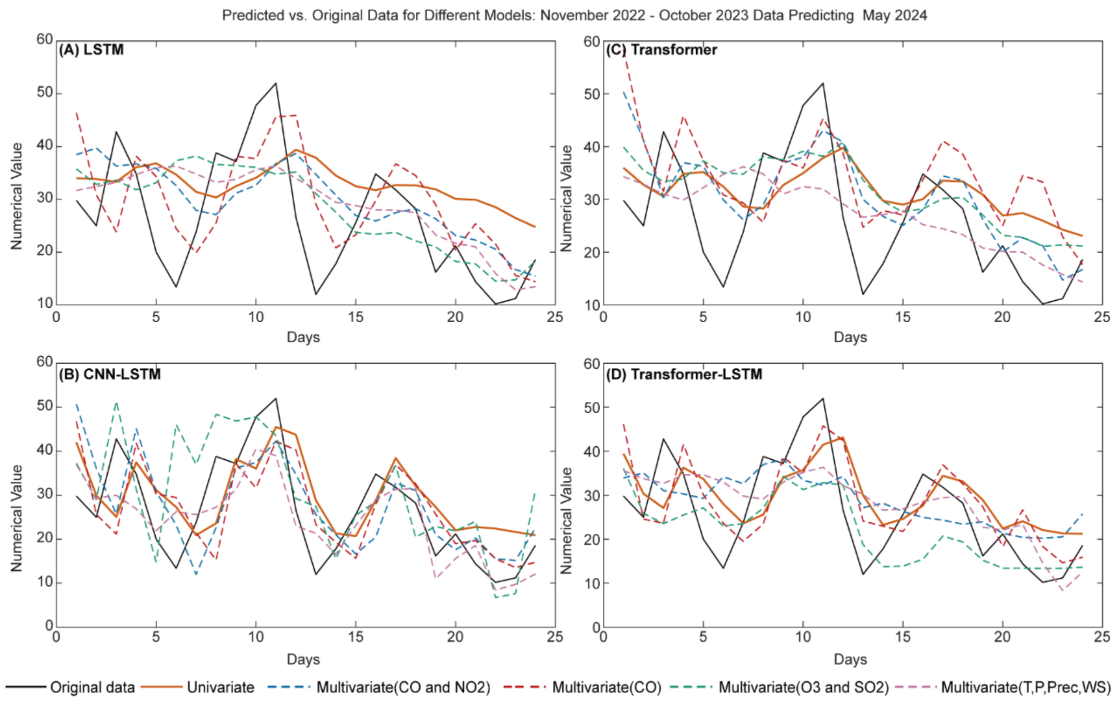


Fig. S14. Predicted vs. Original Data for Different Models Category1-4_Case 7 Short-term monthly predictions

(A)LSTM (B) CNN-LSTM (C) Transformer (D) Transformer-LSTM

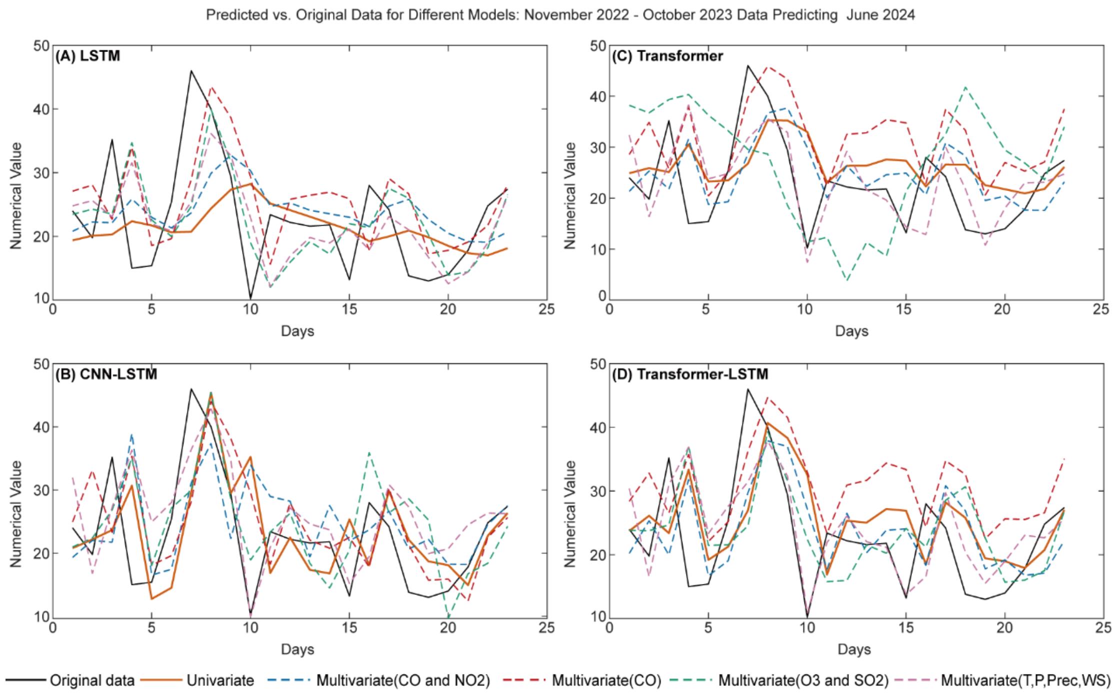


Fig. S15. Predicted vs. Original Data for Different Models Category1-4_Case 8 Short-term monthly predictions

(A)LSTM (B) CNN-LSTM (C) Transformer (D) Transformer-LSTM

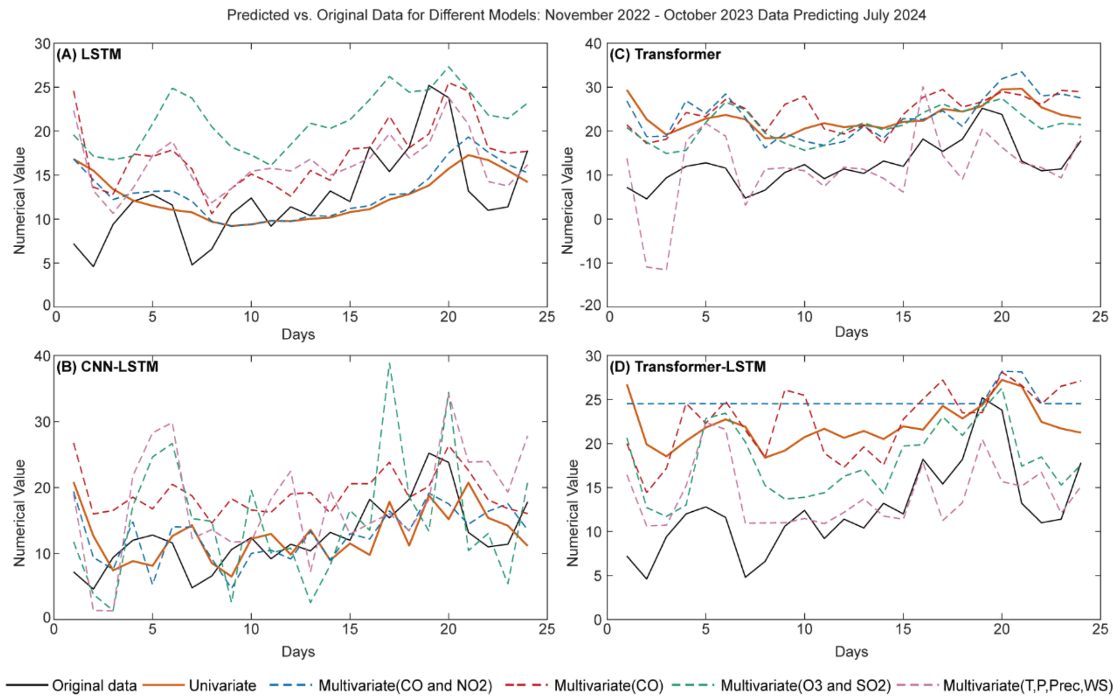


Fig. S16. Predicted vs. Original Data for Different Models Category1-4_Case 9 Short-term monthly predictions

(A)LSTM (B) CNN-LSTM (C) Transformer (D) Transformer-LSTM

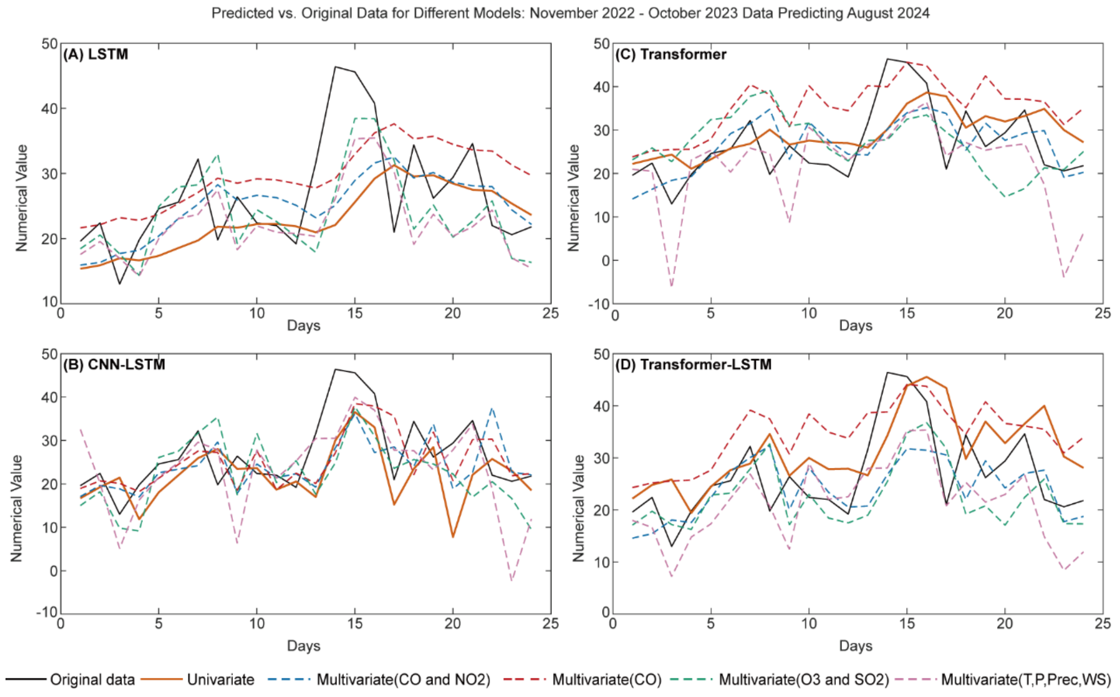


Fig. S17. Predicted vs. Original Data for Different Models Category1-4_Case 10 Short-

term monthly predictions

(A)LSTM (B) CNN-LSTM (C) Transformer (D) Transformer-LSTM

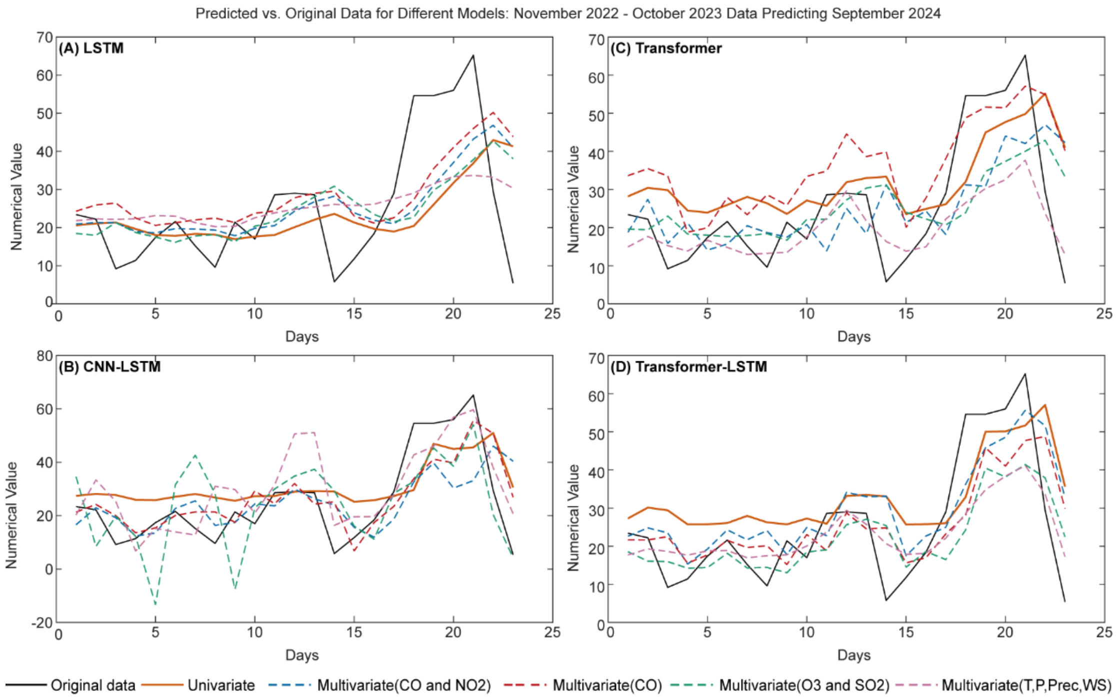


Fig. S18. Predicted vs. Original Data for Different Models Category1-4_Case 11 Short-term monthly predictions

(A)LSTM (B) CNN-LSTM (C) Transformer (D) Transformer-LSTM

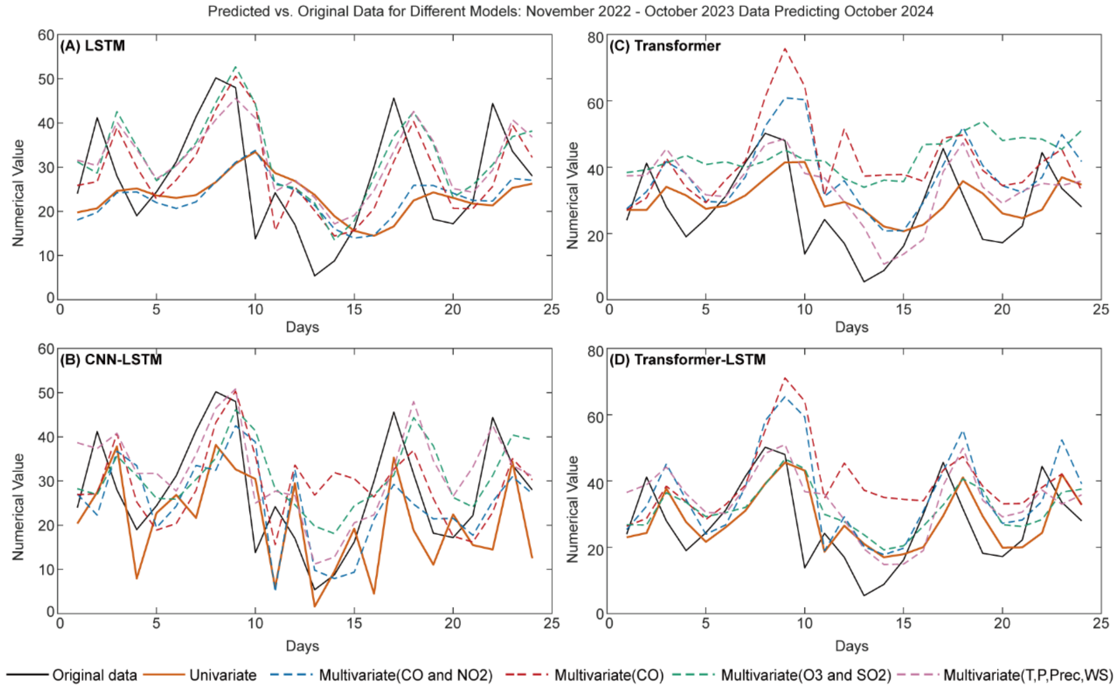

Fig. S19. Predicted vs. Original Data for Different Models Category1-4_Case 12 Short-term monthly predictions

(A)LSTM (B) CNN-LSTM (C) Transformer (D) Transformer-LSTM

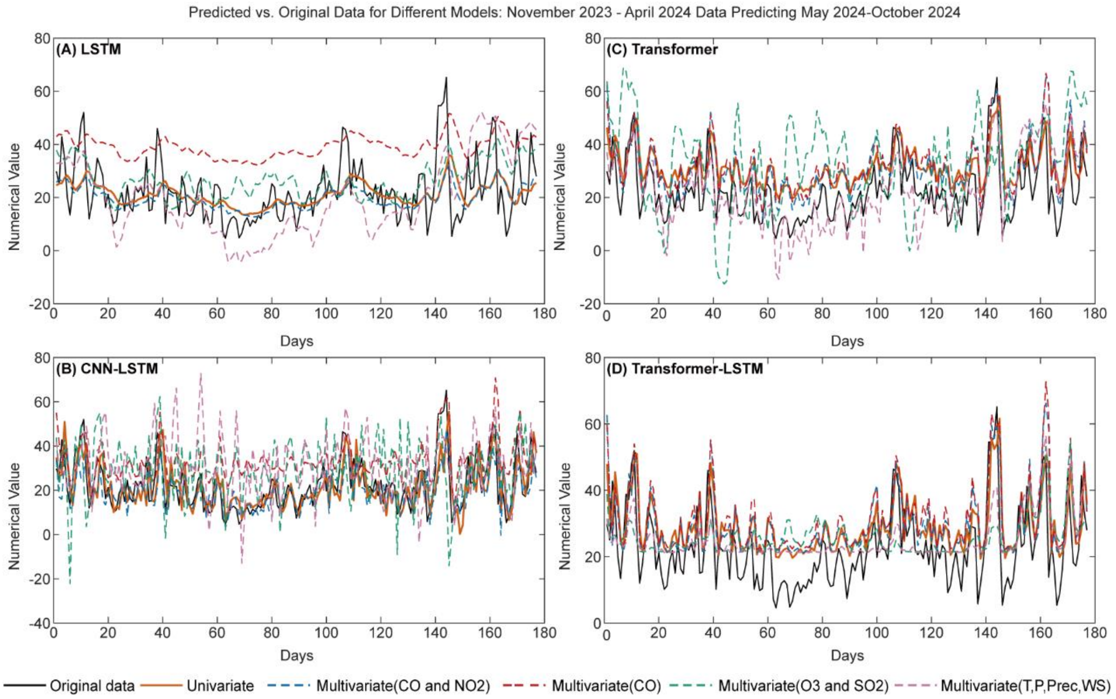


Fig. S20. Predicted vs. Original Data for Different Models Category2-1_Case 1 Seasonal gap predictions

(A)LSTM (B) CNN-LSTM (C) Transformer (D) Transformer-LSTM

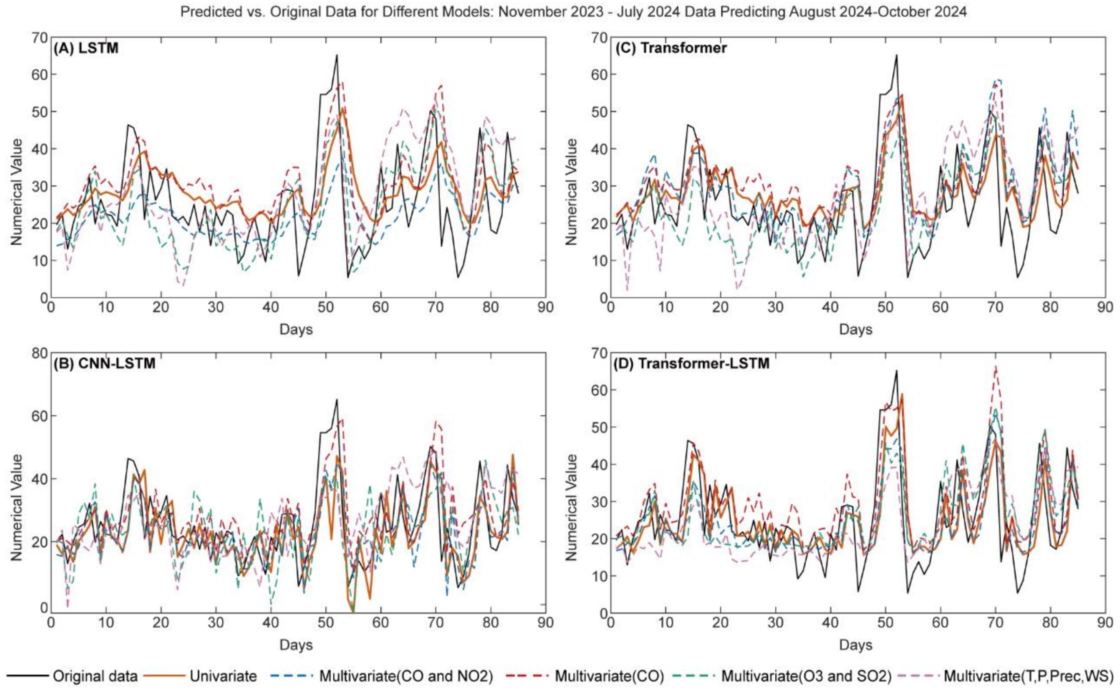


Fig. S21. Predicted vs. Original Data for Different Models Category2-1_Case 2 Seasonal gap predictions

(A)LSTM (B) CNN-LSTM (C) Transformer (D) Transformer-LSTM

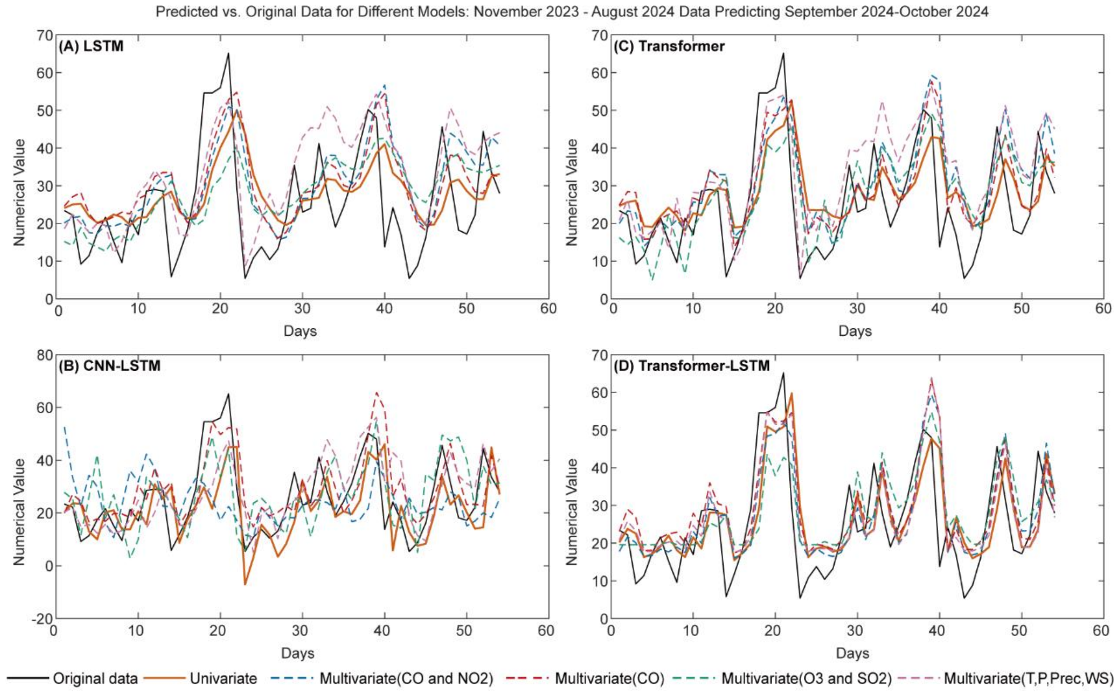


Fig. S22. Predicted vs. Original Data for Different Models Category2-2_Case 1 Short-term predictions with missing months

(A)LSTM (B) CNN-LSTM (C) Transformer (D) Transformer-LSTM

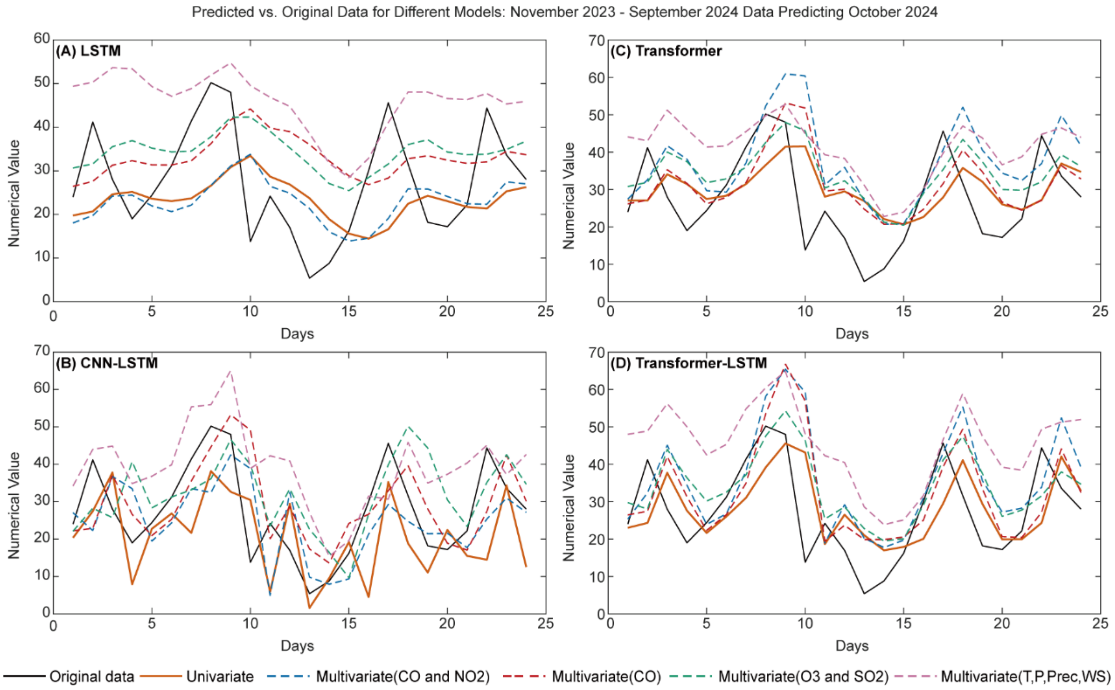


Fig. S23. Predicted vs. Original Data for Different Models Category2-2_Case 2 Short-term predictions with missing months

(A)LSTM (B) CNN-LSTM (C) Transformer (D) Transformer-LSTM

**Table S1. Model performances of Category 1-1 Long-term predictions: Predicting 2023.11- 2024.10 PM2.5 by 2022.11-2023.10 data**

| Variable | Model | $R^2$ | MAE% | RMSE% |
|---|---|---|---|---|
| univariate | LSTM | 0.537 | 0.451 | 0.584 |
| | CNN-LSTM | 0.689 | 0.349 | 0.478 |
| | Transformer | 0.664 | 0.377 | 0.497 |
| | Transformer-LSTM | 0.715 | 0.339 | 0.458 |
| multivariate CO and $NO_2$ | Multi-LSTM | 0.521 | 0.464 | 0.593 |
| | Multi-CNN-LSTM | 0.586 | 0.397 | 0.552 |
| | Multi-Transformer | 0.686 | 0.357 | 0.481 |
| | Multi-Transformer-LSTM | 0.700 | 0.355 | 0.47 |
| multivariate CO | Multi-LSTM | 0.463 | 0.419 | 0.584 |
| | Multi-CNN-LSTM | 0.664 | 0.325 | 0.462 |
| | Multi-Transformer | 0.586 | 0.366 | 0.513 |
| | Multi-Transformer-LSTM | 0.650 | 0.334 | 0.471 |
| multivariate $O_3$ and $SO_2$ | Multi-LSTM | 0.534 | 0.372 | 0.544 |
| | Multi-CNN-LSTM | 0.634 | 0.342 | 0.482 |
| | Multi-Transformer | 0.588 | 0.367 | 0.512 |

| | | | | |
|---|---|---|---|---|
| | Multi-Transformer-LSTM | 0.639 | 0.344 | 0.479 |
| multivariate Temperature, Pressure, Precipitation, Wind speed | Multi-LSTM | 0.675 | 0.317 | 0.453 |
| | Multi-CNN-LSTM | 0.696 | 0.299 | 0.438 |
| | Multi-Transformer | 0.712 | 0.294 | 0.427 |
| | Multi-Transformer-LSTM | 0.734 | 0.277 | 0.410 |

Note: Predicting PM2.5 concentrations for 2023.11–2024.10, based on data spanning 2022.11.1–2023.10.31, and examines how the inclusion of different auxiliary variables improves prediction accuracy.

**Table S2. Models' performances of Category1-2 Mid-term predictions: using 2022.11-2023.10 data**

| Date | Variable | Model | $R^2$ | MAE% | RMSE% |
|---|---|---|---|---|---|
| **Case 1:** Predicting 2023.11-2024.04 | univariate | LSTM | 0.431 | 0.401 | 0.513 |
| | | CNN-LSTM | 0.623 | 0.316 | 0.418 |
| | | Transformer | 0.614 | 0.319 | 0.423 |
| | | Transformer-LSTM | 0.661 | 0.299 | 0.396 |
| | multivariate CO and $NO_2$ | Multi-LSTM | 0.491 | 0.386 | 0.485 |
| | | Multi-CNN-LSTM | 0.488 | 0.358 | 0.487 |
| | | Multi-Transformer | 0.627 | 0.320 | 0.416 |
| | | Multi-Transformer-LSTM | 0.612 | 0.303 | 0.424 |
| | multivariate CO | Multi-LSTM | 0.524 | 0.392 | 0.550 |
| | | Multi-CNN-LSTM | 0.589 | 0.331 | 0.433 |
| | | Multi-Transformer | 0.584 | 0.330 | 0.436 |
| | | Multi-Transformer-LSTM | 0.596 | 0.326 | 0.430 |
| | multivariate $O_3$ and $SO_2$ | Multi-LSTM | 0.484 | 0.366 | 0.486 |
| | | xxMulti-CNN-LSTM | 0.566 | 0.335 | 0.445 |
| | | Multi-Transformer | 0.510 | 0.337 | 0.402 |
| | | Multi-Transformer-LSTM | 0.611 | 0.318 | 0.421 |
| | multivariate T, P, Prec, WS | Multi-LSTM | 0.592 | 0.327 | 0.435 |
| | | Multi-CNN-LSTM | 0.666 | 0.289 | 0.393 |
| | | Multi-Transformer | 0.651 | 0.297 | 0.402 |
| | | Multi-Transformer-LSTM | 0.668 | 0.286 | 0.392 |
| **Case 2:** Predicting 2024.05-2024.10 | univariate | LSTM | 0.280 | 0.328 | 0.429 |
| | | CNN-LSTM | 0.273 | 0.346 | 0.431 |
| | | Transformer | 0.314 | 0.323 | 0.419 |
| | | Transformer-LSTM | 0.348 | 0.316 | 0.408 |
| | multivariate CO and $NO_2$ | Multi -LSTM | 0.204 | 0.342 | 0.452 |
| | | Multi-CNN -LSTM | 0.301 | 0.3311 | 0.423 |
| | | Multi-Transformer | 0.326 | 0.319 | 0.415 |
| | | Multi-Transformer-LSTM | 0.351 | 0.323 | 0.407 |
| | multivariate | Multi- LSTM | 0.251 | 0.368 | 0.493 |

| | | | | | |
|---|---|---|---|---|---|
| | CO | Multi-CNN-LSTM | 0.326 | 0.326 | 0.415 |
| | | Multi-Transformer | 0.244 | 0.348 | 0.439 |
| | | Multi-Transformer-LSTM | 0.331 | 0.325 | 0.413 |
| | multivariate O3 and SO2 | Multi-CNN-LSTM | 0.329 | 0.324 | 0.414 |
| | | Multi-LSTM | 0.268 | 0.336 | 0.433 |
| | | Multi-Transformer | 0.342 | 0.323 | 0.410 |
| | | Multi-Transformer-LSTM | 0.360 | 0.319 | 0.404 |
| | multivariate T, P, Prec, WS | Multi-CNN-LSTM | 0.378 | 0.307 | 0.396 |
| | | Multi-LSTM | 0.459 | 0.287 | 0.369 |
| | | Multi-Transformer | 0.473 | 0.276 | 0.364 |
| | | Multi-Transformer-LSTM | 0.565 | 0.294 | 0.331 |

Note: Predicting PM2.5 concentrations for the periods 2023.11-2024.4 and 2024.5-2024.10, using data from 2022.11.1-2023.10.31, and evaluates the improvement in prediction effectiveness achieved by incorporating different auxiliary variables.

**Table S3. Models' performances of Category 1-3 Short-to-medium-term seasonal predictions**

| Date | Variable | Model | $R^2$ | MAE% | RMSE% |
|---|---|---|---|---|---|
| | univariate | LSTM | 0.427 | 0.359 | 0.454 |
| | | CNN-LSTM | 0.554 | 0.311 | 0.403 |
| | | Transformer | 0.510 | 0.337 | 0.422 |
| | | Transformer-LSTM | 0.609 | 0.304 | 0.377 |
| | multivariate CO and $NO_2$ | Multi-LSTM | 0.367 | 0.381 | 0.48 |
| | | Multi-CNN-LSTM | 0.387 | 0.35 | 0.473 |
| | | Multi-Transformer | 0.559 | 0.324 | 0.401 |
| | | Multi-Transformer-LSTM | 0.604 | 0.303 | 0.38 |
| **Case 1:** Predicting 2023.11-2024.01 | multivariate CO | Multi-LSTM | 0.495 | 0.405 | 0.566 |
| | | Multi-CNN-LSTM | 0.573 | 0.311 | 0.386 |
| | | Multi-Transformer | 0.416 | 0.361 | 0.458 |
| | | Multi-Transformer-LSTM | 0.593 | 0.308 | 0.383 |
| | multivariate $O_3$ and $SO_2$ | Multi-LSTM | 0.531 | 0.322 | 0.411 |
| | | Multi-CNN-LSTM | 0.573 | 0.306 | 0.392 |
| | | Multi-Transformer | 0.518 | 0.334 | 0.399 |
| | | Multi-Transformer-LSTM | 0.610 | 0.302 | 0.375 |
| | multivariate T, P, Prec, WS | Multi-LSTM | 0.634 | 0.287 | 0.373 |
| | | Multi-CNN-LSTM | 0.640 | 0.282 | 0.370 |
| | | Multi-Transformer | 0.648 | 0.285 | 0.366 |
| | | Multi-Transformer-LSTM | 0.679 | 0.269 | 0.349 |
| **Case 2:** | univariate | LSTM | 0.116 | 0.419 | 0.568 |

| | | | | | |
|---|---|---|---|---|---|
| Predicting 2024.02-2024.04 ` | | CNN-LSTM | 0.204 | 0.381 | 0.539 |
| | | Transformer | 0.175 | 0.403 | 0.548 |
| | | Transformer-LSTM | 0.206 | 0.421 | 0.538 |
| | multivariate CO and $NO_2$ | Multi-LSTM | 0.078 | 0.433 | 0.583 |
| | | Multi-CNN-LSTM | 0.185 | 0.398 | 0.549 |
| | | Multi-Transformer | 0.146 | 0.421 | 0.561 |
| | | Multi-Transformer-LSTM | 0.210 | 0.390 | 0.540 |
| | multivariate CO | LSTM | 0.192 | 0.402 | 0.543 |
| | | CNN-LSTM | 0.206 | 0.389 | 0.538 |
| | | Transformer | 0.221 | 0.376 | 0.533 |
| | | Transformer-LSTM | 0.252 | 0.373 | 0.522 |
| | multivariate $O_3$ and $SO_2$ | LSTM | 0.190 | 0.394 | 0.543 |
| | | CNN-LSTM | 0.306 | 0.350 | 0.503 |
| | | Transformer | 0.292 | 0.369 | 0.508 |
| | | Transformer-LSTM | 0.340 | 0.349 | 0.491 |
| | multivariate T, P, Prec, WS | LSTM | 0.272 | 0.369 | 0.524 |
| | | CNN-LSTM | 0.336 | 0.338 | 0.500 |
| | | Transformer | 0.363 | 0.327 | 0.490 |
| | | Transformer-LSTM | 0.418 | 0.310 | 0.468 |
| **Case 3:** Predicting 2024.05-2024.07 | univariate | LSTM | 0.26 | 0.329 | 0.428 |
| | | CNN-LSTM | 0.343 | 0.299 | 0.403 |
| | | Transformer | -0.752 | 0.58 | 0.659 |
| | | Transformer-LSTM | -0.319 | 0.489 | 0.571 |
| | multivariate CO and $NO_2$ | Multi-LSTM | 0.304 | 0.319 | 0.415 |
| | | Multi-CNN-LSTM | 0.317 | 0.319 | 0.411 |
| | | Multi-Transformer | -0.337 | 0.475 | 0.575 |
| | | Multi-Transformer-LSTM | -0.163 | 0.455 | 0.537 |
| | multivariate CO | Multi-LSTM | -0.003 | 0.413 | 0.495 |
| | | Multi-CNN-LSTM | 0.048 | 0.409 | 0.483 |
| | | Multi-Transformer | 0.060 | 0.400 | 0.480 |
| | | Multi-Transformer-LSTM | 0.139 | 0.374 | 0.459 |
| | multivariate O3 and SO2 | Multi-LSTM | 0.108 | 0.396 | 0.467 |
| | | Multi-CNN-LSTM | -0.063 | 0.399 | 0.510 |
| | | Multi-Transformer | 0.233 | 0.354 | 0.433 |
| | | Multi-Transformer-LSTM | 0.323 | 0.332 | 0.407 |
| | multivariate T, P, Prec, WS | Multi-LSTM | 0.247 | 0.353 | 0.429 |
| | | Multi-CNN-LSTM | 0.346 | 0.324 | 0.400 |
| | | Multi-Transformer | 0.452 | 0.285 | 0.366 |
| | | Multi-Transformer-LSTM | 0.438 | 0.308 | 0.371 |
| Case 4: Predicting 2024.08-2024.10 | univariate | LSTM | 0.079 | 0.351 | 0.465 |
| | | CNN-LSTM | 0.132 | 0.374 | 0.451 |
| | | Transformer | 0.128 | 0.352 | 0.452 |
| | | Transformer-LSTM | 0.274 | 0.312 | 0.412 |

| | | | | |
|---|---|---|---|---|
| multivariate CO and $NO_2$ | Multi-LSTM | -0.064 | 0.378 | 0.499 |
| | Multi-CNN-LSTM | 0.208 | 0.338 | 0.431 |
| | Multi-Transformer | 0.207 | 0.335 | 0.431 |
| | Multi-Transformer-LSTM | 0.213 | 0.329 | 0.430 |
| multivariate CO | Multi-LSTM | 0.084 | 0.381 | 0.484 |
| | Multi-CNN-LSTM | 0.121 | 0.379 | 0.454 |
| | Multi-Transformer | 0.215 | 0.332 | 0.429 |
| | Multi-Transformer-LSTM | 0.242 | 0.323 | 0.421 |
| multivariate O3 and SO2 | Multi-LSTM | -0.020 | 0.372 | 0.489 |
| | Multi-CNN-LSTM | 0.022 | 0.377 | 0.479 |
| | Multi-Transformer | 0.264 | 0.321 | 0.415 |
| | Multi-Transformer-LSTM | 0.262 | 0.321 | 0.416 |
| multivariate T, P, Prec, WS | Multi-LSTM | 0.107 | 0.362 | 0.458 |
| | Multi-CNN-LSTM | 0.139 | 0.339 | 0.450 |
| | Multi-Transformer | 0.245 | 0.336 | 0.421 |
| | Multi-Transformer-LSTM | 0.309 | 0.309 | 0.402 |

Note: Predicting PM2.5 concentrations across four quarters, based on data spanning 2022.11.1–2023.10.31, and examines the improvement in prediction effectiveness achieved by incorporating different auxiliary variables.

**Table S4. Models' performances of Category 1-4 Short-term monthly predictions**

| Date | Variable | Model | $R^2$ | MAE% | RMSE% |
|---|---|---|---|---|---|
| Case 1: Predicting 2023.11 | univariate | LSTM | 0.322 | 0.367 | 0.463 |
| | | CNN-LSTM | 0.329 | 0.380 | 0.460 |
| | | Transformer | 0.297 | 0.370 | 0.471 |
| | | Transformer-LSTM | 0.399 | 0.348 | 0.435 |
| | multivariate CO and $NO_2$ | Multi-LSTM | 0.226 | 0.394 | 0.505 |
| | | Multi-CNN-LSTM | 0.430 | 0.335 | 0.433 |
| | | Multi-Transformer | 0.270 | 0.387 | 0.490 |
| | | Multi-Transformer-LSTM | 0.442 | 0.344 | 0.429 |
| | multivariate CO | Multi-LSTM | 0.341 | 0.360 | 0.456 |
| | | Multi-CNN-LSTM | 0.414 | 0.336 | 0.430 |
| | | Multi-Transformer | 0.399 | 0.349 | 0.435 |
| | | Multi-Transformer-LSTM | 0.367 | 0.353 | 0.447 |
| | multivariate O3 and SO2 | Multi-LSTM | 0.184 | 0.396 | 0.507 |
| | | Multi-CNN-LSTM | 0.239 | 0.377 | 0.490 |
| | | Multi-Transformer | 0.288 | 0.365 | 0.474 |
| | | Multi-Transformer-LSTM | 0.331 | 0.333 | 0.459 |
| | multivariate T, P, Prec, WS | Multi-LSTM | 0.365 | 0.352 | 0.462 |
| | | Multi-CNN-LSTM | 0.504 | 0.332 | 0.408 |
| | | Multi-Transformer | 0.439 | 0.344 | 0.434 |
| | | Multi-Transformer-LSTM | 0.518 | 0.321 | 0.402 |
| **Case 2:** Predicting 2023.12 | univariate | LSTM | 0.555 | 0.322 | 0.383 |
| | | CNN-LSTM | 0.523 | 0.328 | 0.397 |
| | | Transformer | 0.567 | 0.320 | 0.378 |
| | | Transformer-LSTM | 0.584 | 0.312 | 0.370 |
| | multivariate CO and $NO_2$ | Multi-LSTM | 0.565 | 0.350 | 0.416 |
| | | Multi-CNN-LSTM | 0.506 | 0.392 | 0.443 |
| | | Multi-Transformer | 0.564 | 0.350 | 0.416 |
| | | Multi-Transformer-LSTM | 0.580 | 0.321 | 0.380 |
| | multivariate CO | LSTM | 0.538 | 0.333 | 0.391 |
| | | CNN-LSTM | 0.540 | 0.340 | 0.390 |
| | | Transformer | 0.574 | 0.305 | 0.375 |
| | | Transformer-LSTM | 0.591 | 0.313 | 0.367 |
| | multivariate $O_3$ and $SO_2$ | LSTM | 0.591 | 0.298 | 0.368 |
| | | CNN-LSTM | 0.495 | 0.327 | 0.408 |
| | | Transformer | 0.580 | 0.321 | 0.372 |
| | | Transformer-LSTM | 0.605 | 0.312 | 0.361 |
| | multivariate T, P, Prec, WS | LSTM | 0.586 | 0.320 | 0.405 |
| | | CNN-LSTM | 0.586 | 0.320 | 0.405 |
| | | Transformer | 0.606 | 0.312 | 0.396 |
| | | Transformer-LSTM | 0.623 | 0.313 | 0.387 |
| Case 3: Predicting 2024.01 | univariate | LSTM | 0.417 | 0.392 | 0.478 |
| | | CNN-LSTM | 0.600 | 0.274 | 0.397 |
| | | Transformer | 0.575 | 0.319 | 0.408 |
| | | Transformer-LSTM | 0.651 | 0.294 | 0.370 |
| | multivariate CO and $NO_2$ | Multi-LSTM | 0.373 | 0.385 | 0.496 |
| | | Multi-CNN-LSTM | 0.422 | 0.324 | 0.476 |

| | | | | | |
|---|---|---|---|---|---|
| | | Multi-Transformer | 0.592 | 0.302 | 0.400 |
| | | Multi-Transformer-LSTM | 0.613 | 0.282 | 0.390 |
| | multivariate CO | Multi-LSTM | 0.451 | 0.343 | 0.482 |
| | | Multi-CNN-LSTM | 0.550 | 0.288 | 0.412 |
| | | Multi-Transformer | 0.506 | 0.340 | 0.431 |
| | | Multi-Transformer-LSTM | 0.620 | 0.292 | 0.378 |
| | multivariate $O_3$ and $SO_2$ | Multi-LSTM | 0.607 | 0.307 | 0.385 |
| | | Multi-CNN-LSTM | 0.547 | 0.287 | 0.380 |
| | | Multi-Transformer | 0.548 | 0.324 | 0.413 |
| | | Multi-Transformer-LSTM | 0.612 | 0.294 | 0.382 |
| | multivariate T, P, Prec, WS | Multi-LSTM | 0.665 | 0.257 | 0.327 |
| | | Multi-CNN-LSTM | 0.643 | 0.245 | 0.337 |
| | | Multi-Transformer | 0.671 | 0.242 | 0.324 |
| | | Multi-Transformer-LSTM | 0.701 | 0.226 | 0.309 |
| **Case 4:** Predicting 2024.02 | univariate | LSTM | -0.150 | 0.532 | 0.670 |
| | | Transformer | 0.242 | 0.424 | 0.544 |
| | | CNN-LSTM | 0.148 | 0.422 | 0.576 |
| | | Transformer-LSTM | 0.165 | 0.395 | 0.571 |
| | multivariate CO and $NO_2$ | Multi-LSTM | -0.070 | 0.512 | 0.646 |
| | | Multi-CNN-LSTM | 0.296 | 0.402 | 0.524 |
| | | Multi-Transformer | 0.115 | 0.456 | 0.588 |
| | | Multi-Transformer-LSTM | 0.113 | 0.440 | 0.588 |
| | multivariate CO | Multi-LSTM | 0.095 | 0.416 | 0.581 |
| | | Multi-CNN-LSTM | 0.202 | 0.169 | 0.211 |
| | | Multi-Transformer | 0.059 | 0.389 | 0.592 |
| | | Multi-Transformer-LSTM | 0.091 | 0.382 | 0.582 |
| | multivariate $O_3$ and $SO_2$ | Multi-LSTM | 0.171 | 0.411 | 0.617 |
| | | Multi-CNN-LSTM | -0.046 | 0.554 | 0.693 |
| | | Multi-Transformer | 0.039 | 0.491 | 0.664 |
| | | Multi-Transformer-LSTM | 0.036 | 0.475 | 0.665 |
| | multivariate T, P, Prec, WS | Multi-LSTM | 0.205 | 0.397 | 0.604 |
| | | Multi-CNN-LSTM | 0.034 | 0.481 | 0.666 |
| | | Multi-Transformer | 0.135 | 0.463 | 0.630 |
| | | Multi-Transformer-LSTM | 0.224 | 0.391 | 0.538 |
| **Case 5:** Predicting 2024.03 | univariate | LSTM | 0.087 | 0.359 | 0.432 |
| | | CNN-LSTM | 0.136 | 0.344 | 0.421 |
| | | Transformer | 0.032 | 0.375 | 0.445 |
| | | Transformer-LSTM | 0.078 | 0.351 | 0.434 |
| | multivariate CO and $NO_2$ | Multi-LSTM | 0.173 | 0.320 | 0.380 |
| | | Multi-CNN-LSTM | 0.138 | 0.335 | 0.429 |
| | | Multi-Transformer | 0.133 | 0.275 | 0.389 |
| | | Multi-Transformer-LSTM | 0.094 | 0.278 | 0.398 |
| | multivariate | Multi-LSTM | 0.178 | 0.329 | 0.410 |

| | | | | | |
|---|---|---|---|---|---|
| | CO | Multi-CNN-LSTM | 0.008 | 0.370 | 0.451 |
| | | Multi-Transformer | -0.036 | 0.349 | 0.461 |
| | | Multi-Transformer-LSTM | 0.024 | 0.346 | 0.447 |
| | multivariate $O_3$ and $SO_2$ | Multi-LSTM | 0.253 | 0.299 | 0.361 |
| | | Multi-CNN-LSTM | 0.121 | 0.315 | 0.392 |
| | | Multi-Transformer | 0.203 | 0.313 | 0.373 |
| | | Multi-Transformer-LSTM | 0.162 | 0.312 | 0.382 |
| | multivariate T, P, Prec, WS | Multi-LSTM | 0.105 | 0.305 | 0.395 |
| | | Multi-CNN-LSTM | 0.547 | 0.216 | 0.281 |
| | | Multi-Transformer | 0.469 | 0.226 | 0.304 |
| | | Multi-Transformer-LSTM | 0.612 | 0.192 | 0.260 |
| **Case 6:** Predicting 2024.04 | univariate | LSTM | -0.105 | 0.492 | 0.627 |
| | | CNN-LSTM | 0.160 | 0.439 | 0.559 |
| | | Transformer | 0.085 | 0.471 | 0.583 |
| | | Transformer-LSTM | 0.117 | 0.445 | 0.573 |
| | multivariate CO and $NO_2$ | Multi-LSTM | 0.026 | 0.455 | 0.588 |
| | | Multi-CNN-LSTM | 0.026 | 0.474 | 0.602 |
| | | Multi-Transformer | 0.191 | 0.414 | 0.548 |
| | | Multi-Transformer-LSTM | 0.174 | 0.444 | 0.554 |
| | multivariate CO | LSTM | 0.091 | 0.438 | 0.568 |
| | | CNN-LSTM | 0.084 | 0.430 | 0.571 |
| | | Transformer | 0.162 | 0.424 | 0.546 |
| | | Transformer-LSTM | 0.161 | 0.424 | 0.546 |
| | multivariate $O_3$ and $SO_2$ | LSTM | 0.221 | 0.418 | 0.543 |
| | | CNN-LSTM | 0.125 | 0.421 | 0.575 |
| | | Transformer | -0.123 | 0.514 | 0.652 |
| | | Transformer-LSTM | -0.104 | 0.509 | 0.646 |
| | multivariate T, P, Prec, WS | LSTM | 0.134 | 0.462 | 0.572 |
| | | CNN-LSTM | 0.303 | 0.378 | 0.513 |
| | | Transformer | 0.330 | 0.384 | 0.504 |
| | | Transformer-LSTM | 0.361 | 0.371 | 0.492 |
| Case 7: Predicting 2024.05 | univariate | LSTM | 0.004 | 0.364 | 0.434 |
| | | CNN-LSTM | 0.242 | 0.322 | 0.379 |
| | | Transformer | 0.020 | 0.373 | 0.431 |
| | | Transformer-LSTM | 0.233 | 0.321 | 0.381 |
| | multivariate CO and $NO_2$ | Multi-LSTM | 0.098 | 0.348 | 0.413 |
| | | Multi-CNN-LSTM | 0.222 | 0.329 | 0.384 |
| | | Multi-Transformer | 0.161 | 0.328 | 0.398 |
| | | Multi-Transformer-LSTM | 0.248 | 0.320 | 0.377 |
| | multivariate CO | Multi-LSTM | 0.190 | 0.327 | 0.391 |
| | | Multi-CNN-LSTM | 0.202 | 0.330 | 0.414 |
| | | Multi-Transformer | 0.152 | 0.338 | 0.400 |
| | | Multi-Transformer-LSTM | 0.245 | 0.321 | 0.378 |

| | | | | | |
|---|---|---|---|---|---|
| | multivariate O3 and SO2 | Multi-LSTM | 0.234 | 0.305 | 0.381 |
| | | Multi-CNN-LSTM | 0.248 | 0.282 | 0.377 |
| | | Multi-Transformer | 0.134 | 0.330 | 0.405 |
| | | Multi-Transformer-LSTM | 0.263 | 0.301 | 0.373 |
| | multivariate T, P, Prec, WS | Multi-LSTM | 0.273 | 0.293 | 0.371 |
| | | Multi-CNN-LSTM | 0.514 | 0.243 | 0.303 |
| | | Multi-Transformer | 0.266 | 0.313 | 0.373 |
| | | Multi-Transformer-LSTM | 0.356 | 0.286 | 0.349 |
| **Case 8:** Predicting 2024.06 | univariate | LSTM | -0.093 | 0.307 | 0.408 |
| | | Transformer | -0.059 | 0.306 | 0.402 |
| | | CNN-LSTM | -0.113 | 0.323 | 0.412 |
| | | Transformer-LSTM | -0.150 | 0.322 | 0.419 |
| | multivariate CO and $NO_2$ | Multi-LSTM | -0.116 | 0.300 | 0.413 |
| | | Multi-CNN-LSTM | -0.044 | 0.313 | 0.399 |
| | | Multi-Transformer | -0.063 | 0.327 | 0.403 |
| | | Multi-Transformer-LSTM | -0.004 | 0.322 | 0.391 |
| | multivariate CO | Multi-LSTM | -0.154 | 0.335 | 0.410 |
| | | Multi-CNN-LSTM | 0.202 | 0.382 | 0.478 |
| | | Multi-Transformer | -1.006 | 0.460 | 0.541 |
| | | Multi-Transformer-LSTM | -0.767 | 0.430 | 0.508 |
| | multivariate $O_3$ and $SO_2$ | Multi-LSTM | 0.002 | 0.305 | 0.394 |
| | | Multi-CNN-LSTM | 0.141 | 0.282 | 0.365 |
| | | Multi-Transformer | -1.682 | 0.554 | 0.646 |
| | | Multi-Transformer-LSTM | -1.254 | 0.492 | 0.592 |
| | multivariate T, P, Prec, WS | Multi-LSTM | 0.099 | 0.294 | 0.374 |
| | | Multi-CNN-LSTM | 0.255 | 0.268 | 0.340 |
| | | Multi-Transformer | 0.164 | 0.268 | 0.361 |
| | | Multi-Transformer-LSTM | 0.354 | 0.223 | 0.317 |
| **Case 9:** Predicting 2024.07 | univariate | LSTM | -0.040 | 0.329 | 0.417 |
| | | CNN-LSTM | -0.274 | 0.382 | 0.461 |
| | | Transformer | -3.074 | 0.723 | 0.808 |
| | | Transformer-LSTM | -3.281 | 0.754 | 0.828 |
| | multivariate CO and $NO_2$ | Multi-LSTM | 0.025 | 0.300 | 0.367 |
| | | Multi-CNN-LSTM | 0.006 | 0.345 | 0.408 |
| | | Multi-Transformer | -0.250 | 0.331 | 0.416 |
| | | Multi-Transformer-LSTM | -0.255 | 0.330 | 0.417 |
| | multivariate CO | Multi-LSTM | -0.656 | 0.407 | 0.515 |
| | | Multi-CNN-LSTM | -1.592 | 0.558 | 0.644 |
| | | Multi-Transformer | -5.128 | 0.929 | 0.991 |
| | | Multi-Transformer-LSTM | -3.642 | 0.807 | 0.862 |
| | multivariate O3 and SO2 | Multi-LSTM | -1.392 | 0.506 | 0.575 |
| | | Multi-CNN-LSTM | -1.840 | 0.515 | 0.627 |
| | | Multi-Transformer | -2.702 | 0.690 | 0.770 |

| | | | | | |
|---|---|---|---|---|---|
| | | Multi-Transformer-LSTM | -0.863 | 0.438 | 0.546 |
| | multivariate T, P, Prec, WS | Multi-LSTM | -0.288 | 0.340 | 0.422 |
| | | Multi-CNN-LSTM | -2.082 | 0.557 | 0.653 |
| | | Multi-Transformer | -1.103 | 0.386 | 0.540 |
| | | Multi-Transformer-LSTM | 0.058 | 0.284 | 0.361 |
| **Case 10:** Predicting 2024.08 | univariate | LSTM | -0.052 | 0.249 | 0.330 |
| | | CNN-LSTM | -0.003 | 0.259 | 0.322 |
| | | Transformer | 0.201 | 0.225 | 0.287 |
| | | Transformer-LSTM | -0.065 | 0.252 | 0.332 |
| | multivariate CO and $NO_2$ | Multi-LSTM | -0.014 | 0.265 | 0.324 |
| | | Multi-CNN-LSTM | 0.185 | 0.229 | 0.290 |
| | | Multi-Transformer | 0.240 | 0.228 | 0.280 |
| | | Multi-Transformer-LSTM | 0.142 | 0.243 | 0.298 |
| | multivariate CO | LSTM | 0.051 | 0.246 | 0.307 |
| | | CNN-LSTM | 0.184 | 0.225 | 0.284 |
| | | Transformer | -0.636 | 0.341 | 0.402 |
| | | Transformer-LSTM | -0.441 | 0.321 | 0.378 |
| | multivariate O3 and SO2 | LSTM | 0.118 | 0.249 | 0.325 |
| | | CNN-LSTM | -0.211 | 0.304 | 0.381 |
| | | Transformer | -0.279 | 0.326 | 0.392 |
| | | Transformer-LSTM | -0.265 | 0.304 | 0.389 |
| | multivariate T, P, Prec, WS | LSTM | 0.096 | 0.258 | 0.299 |
| | | CNN-LSTM | 0.153 | 0.232 | 0.290 |
| | | Transformer | 0.004 | 0.267 | 0.314 |
| | | Transformer-LSTM | 0.159 | 0.265 | 0.318 |
| **Case 11:** Predicting 2024.09 | univariate | LSTM | 0.098 | 0.470 | 0.632 |
| | | CNN-LSTM | 0.307 | 0.443 | 0.542 |
| | | Transformer | 0.222 | 0.471 | 0.587 |
| | | Transformer-LSTM | 0.435 | 0.358 | 0.489 |
| | multivariate CO and $NO_2$ | Multi-LSTM | 0.016 | 0.435 | 0.599 |
| | | Multi-CNN-LSTM | 0.125 | 0.323 | 0.407 |
| | | Multi-Transformer | 0.195 | 0.470 | 0.584 |
| | | Multi-Transformer-LSTM | 0.483 | 0.357 | 0.468 |
| | multivariate CO | Multi-LSTM | 0.194 | 0.432 | 0.584 |
| | | Multi-CNN-LSTM | 0.196 | 0.443 | 0.612 |
| | | Multi-Transformer | 0.133 | 0.490 | 0.606 |
| | | Multi-Transformer-LSTM | 0.491 | 0.347 | 0.464 |
| | multivariate $O_3$ and $SO_2$ | Multi-LSTM | 0.121 | 0.463 | 0.610 |
| | | Multi-CNN-LSTM | 0.159 | 0.504 | 0.626 |
| | | Multi-Transformer | 0.267 | 0.425 | 0.557 |
| | | Multi-Transformer-LSTM | 0.442 | 0.371 | 0.486 |
| | multivariate T, P, Prec, | Multi-LSTM | 0.277 | 0.416 | 0.553 |
| | | Multi-CNN-LSTM | 0.506 | 0.326 | 0.458 |

| | | | | | |
|---|---|---|---|---|---|
| | WS | Multi-Transformer | 0.472 | 0.361 | 0.471 |
| | | Multi-Transformer-LSTM | 0.596 | 0.316 | 0.414 |
| **Case 12:** Predicting 2024.10 | univariate | LSTM | -0.154 | 0.387 | 0.486 |
| | | Transformer | 0.331 | 0.295 | 0.370 |
| | | CNN-LSTM | 0.106 | 0.358 | 0.427 |
| | | Transformer-LSTM | 0.144 | 0.337 | 0.418 |
| | multivariate CO and $NO_2$ | Multi-LSTM | 0.083 | 0.376 | 0.433 |
| | | Multi-CNN-LSTM | -0.149 | 0.403 | 0.485 |
| | | Multi-Transformer | -0.176 | 0.407 | 0.490 |
| | | Multi-Transformer-LSTM | 0.185 | 0.342 | 0.408 |
| | multivariate CO | Multi-LSTM | 0.202 | 0.315 | 0.395 |
| | | Multi-CNN-LSTM | 0.113 | 0.341 | 0.417 |
| | | Multi-Transformer | -1.523 | 0.550 | 0.703 |
| | | Multi-Transformer-LSTM | -1.157 | 0.499 | 0.650 |
| | multivariate O3 and SO2 | Multi-LSTM | 0.101 | 0.369 | 0.455 |
| | | Multi-CNN-LSTM | 0.397 | 0.382 | 0.503 |
| | | Multi-Transformer | -1.549 | 0.660 | 0.765 |
| | | Multi-Transformer-LSTM | -1.310 | 0.634 | 0.728 |
| | multivariate T, P, Prec, WS | Multi-LSTM | 0.176 | 0.361 | 0.435 |
| | | Multi-CNN-LSTM | 0.285 | 0.300 | 0.357 |
| | | Multi-Transformer | 0.109 | 0.373 | 0.452 |
| | | Multi-Transformer-LSTM | 0.191 | 0.357 | 0.432 |

Note: Predicting PM2.5 concentrations for individual months, based on data spanning 2022.11.1–2023.10.31, and examines the improvement in prediction accuracy achieved by incorporating different auxiliary variables.

**Table S5. Models' performances of Category 2-1 Seasonal Gap Predictions**

| Date | Variable | Model | $R^2$ | MAE% | RMSE% |
|---|---|---|---|---|---|
| Case 1 Predicting 2024.05-2024.10 | univariate | LSTM | 0.124 | 0.350 | 0.474 |
| | | CNN-LSTM | 0.271 | 0.339 | 0.433 |
| | | Transformer | -0.193 | 0.471 | 0.554 |
| | | Transformer-LSTM | 0.065 | 0.403 | 0.490 |
| | multivariate CO and $NO_2$ | Multi-LSTM | 0.145 | 0.346 | 0.469 |
| | | Multi-CNN-LSTM | 0.217 | 0.334 | 0.448 |
| | | Multi-Transformer | -0.169 | 0.439 | 0.548 |
| | | Multi-Transformer-LSTM | -0.018 | 0.417 | 0.511 |
| | multivariate CO | Multi-LSTM | -1.992 | 0.790 | 0.874 |
| | | Multi-CNN-LSTM | -0.606 | 0.545 | 0.640 |
| | | Multi-Transformer | -0.305 | 0.477 | 0.577 |
| | | Multi-Transformer-LSTM | -0.241 | 0.461 | 0.563 |
| | multivariate O3 | Multi-LSTM | -0.090 | 0.424 | 0.528 |

| | | | | | |
|---|---|---|---|---|---|
| | and SO2 | Multi-CNN-LSTM | -0.923 | 0.565 | 0.701 |
| | | Multi-Transformer | 0.074 | 0.394 | 0.486 |
| | | Multi-Transformer-LSTM | 0.008 | 0.391 | 0.503 |
| | multivariate T, P, Prec, WS | Multi-LSTM | -0.334 | 0.477 | 0.584 |
| | | Multi-CNN-LSTM | -1.160 | 0.582 | 0.743 |
| | | Multi-Transformer | 0.245 | 0.335 | 0.245 |
| | | Multi-Transformer-LSTM | 0.333 | 0.327 | 0.333 |
| Case 2: Predicting 2024.08-2024.10 | univariate | LSTM | 0.042 | 0.364 | 0.474 |
| | | CNN-LSTM | 0.227 | 0.343 | 0.428 |
| | | Transformer | 0.244 | 0.326 | 0.423 |
| | | Transformer-LSTM | 0.316 | 0.311 | 0.403 |
| | multivariate CO and $NO_2$ | Multi-LSTM | 0.050 | 0.358 | 0.475 |
| | | Multi-CNN-LSTM | 0.266 | 0.328 | 0.417 |
| | | Multi-Transformer | 0.204 | 0.332 | 0.434 |
| | | Multi-Transformer-LSTM | 0.268 | 0.310 | 0.414 |
| | multivariate CO | Multi-LSTM | 0.009 | 0.365 | 0.482 |
| | | Multi-CNN-LSTM | 0.208 | 0.327 | 0.431 |
| | | Multi-Transformer | 0.110 | 0.353 | 0.457 |
| | | Multi-Transformer-LSTM | 0.246 | 0.311 | 0.421 |
| | multivariate O3 and SO2 | Multi-LSTM | 0.009 | 0.365 | 0.482 |
| | | Multi-CNN-LSTM | 0.208 | 0.327 | 0.431 |
| | | Multi-Transformer | 0.103 | 0.365 | 0.458 |
| | | Multi-Transformer-LSTM | 0.264 | 0.312 | 0.415 |
| | multivariate T, P, Prec, WS | Multi-LSTM | 0.044 | 0.365 | 0.473 |
| | | Multi-CNN-LSTM | 0.229 | 0.340 | 0.425 |
| | | Multi-Transformer | 0.144 | 0.355 | 0.448 |
| | | Multi-Transformer-LSTM | 0.350 | 0.317 | 0.390 |

Note: Case 1 Train on winter and spring to predict summer and autumn. Case 2 Train on winter, spring, and summer to predict autumn.

**Table S6. Models' performances of Category 2-2 Short-Term Predictions with Missing Months**

| Date | Variable | Model | $R^2$ | MAE% | RMSE% |
|---|---|---|---|---|---|
| Case 1: Predicting 2024.09-2024.10 | univariate | LSTM | 0.050 | 0.430 | 0.542 |
| | | CNN-LSTM | 0.196 | 0.387 | 0.503 |
| | | Transformer | 0.256 | 0.383 | 0.484 |
| | | Transformer-LSTM | 0.344 | 0.350 | 0.455 |
| | multivariate CO and $NO_2$ | Multi-LSTM | 0.014 | 0.434 | 0.553 |
| | | Multi-CNN-LSTM | 0.267 | 0.360 | 0.476 |
| | | Multi-Transformer | 0.215 | 0.377 | 0.493 |
| | | Multi-Transformer-LSTM | 0.350 | 0.342 | 0.449 |

| | | | | | |
|---|---|---|---|---|---|
| | multivariate CO | Multi-LSTM | 0.049 | 0.421 | 0.543 |
| | | Multi-CNN-LSTM | 0.266 | 0.360 | 0.477 |
| | | Multi-Transformer | 0.314 | 0.350 | 0.461 |
| | | Multi-Transformer-LSTM | 0.309 | 0.364 | 0.463 |
| | multivariate O3 and SO2 | Multi-LSTM | 0.019 | 0.449 | 0.551 |
| | | Multi-CNN-LSTM | 0.220 | 0.391 | 0.491 |
| | | Multi-Transformer | 0.228 | 0.388 | 0.489 |
| | | Multi-Transformer-LSTM | 0.320 | 0.358 | 0.459 |
| | multivariate T, P, Prec, WS | Multi-LSTM | 0.108 | 0.406 | 0.525 |
| | | Multi-CNN-LSTM | 0.350 | 0.362 | 0.448 |
| | | Multi-Transformer | 0.232 | 0.376 | 0.488 |
| | | Multi-Transformer-LSTM | 0.379 | 0.352 | 0.439 |
| **Case 2:** Predicting 2024.10 | univariate | LSTM | -0.201 | 0.398 | 0.495 |
| | | CNN-LSTM | -0.116 | 0.392 | 0.477 |
| | | Transformer | 0.103 | 0.357 | 0.428 |
| | | Transformer-LSTM | 0.172 | 0.336 | 0.411 |
| | multivariate CO and $NO_2$ | Multi-LSTM | -0.134 | 0.387 | 0.481 |
| | | Multi-CNN-LSTM | 0.133 | 0.342 | 0.421 |
| | | Multi-Transformer | -0.578 | 0.445 | 0.568 |
| | | Multi-Transformer-LSTM | -0.380 | 0.404 | 0.531 |
| | multivariate CO | Multi-LSTM | -0.418 | 0.429 | 0.527 |
| | | Multi-CNN-LSTM | 0.167 | 0.313 | 0.404 |
| | | Multi-Transformer | -0.056 | 0.358 | 0.455 |
| | | Multi-Transformer-LSTM | -0.202 | 0.368 | 0.485 |
| | multivariate O3 and SO2 | Multi-LSTM | -0.261 | 0.428 | 0.497 |
| | | Multi-CNN-LSTM | -0.042 | 0.353 | 0.452 |
| | | Multi-Transformer | -0.060 | 0.377 | 0.456 |
| | | Multi-Transformer-LSTM | 0.033 | 0.345 | 0.435 |
| | multivariate T, P, Prec, WS | Multi-LSTM | -2.056 | 0.672 | 0.774 |
| | | Multi-CNN-LSTM | -0.426 | 0.463 | 0.528 |
| | | Multi-Transformer | -0.860 | 0.506 | 0.604 |
| | | Multi-Transformer-LSTM | -1.690 | 0.650 | 0.726 |

Note: Case 1 Train on the first 10 months to predict the next 2 months. Case 2 Train on the first 11 months to predict the final month.

**Table S7. Model Performance metrics of Transformer-LSTM with meteorological factors in Urumqi and Shijiazhuang**

| Variable | Training period | Forecasting Horizon | $R^2$ | MAE% | RMSE% |
|---|---|---|---|---|---|
| | 2022.11- | Full year 2023.11-2024.10 | 0.627 | 0.288 | 0.468 |

| | | | | | |
|---|---|---|---|---|---|
| Urumqi | 2023.10 | First half-year 2023.11-2024.04 | 0.539 | 0.360 | 0.563 |
| | | First quarter 2023.11-2024.01 | 0.596 | 0.346 | 0.536 |
| Hangzhou | 2022.11-2023.10 | Full year 2023.11-2024.10 | 0.614 | 0.331 | 0.514 |
| | | First half-year 2023.11-2024.04 | 0.571 | 0.335 | 0.552 |
| | | First quarter 2023.11-2024.01 | 0.625 | 0.326 | 0.507 |

Note: Using Transformer-LSTM to predict results for Urumqi and Hangzhou across different time periods, respectively.
